# Precise Correction of *Lhcgr* Mutation in Stem Leydig Cells by Prime Editing Rescues Hereditary Primary Hypogonadism in Mice

**DOI:** 10.1002/advs.202300993

**Published:** 2023-09-11

**Authors:** Kai Xia, Fulin Wang, Zhipeng Tan, Suyuan Zhang, Xingqiang Lai, Wangsheng Ou, Cuifeng Yang, Hong Chen, Hao Peng, Peng Luo, Anqi Hu, Xiang'an Tu, Tao Wang, Qiong Ke, Chunhua Deng, Andy Peng Xiang

**Affiliations:** ^1^ Center for Stem Cell Biology and Tissue Engineering Key Laboratory for Stem Cells and Tissue Engineering Ministry of Education National‐Local Joint Engineering Research Center for Stem Cells and Regenerative Medicine Zhongshan School of Medicine Sun Yat‐sen University Guangzhou Guangdong 510080 China; ^2^ Department of Urology and Andrology The First Affiliated Hospital Sun Yat‐sen University Guangzhou Guangdong 510080 China; ^3^ Cardiovascular Department The Eighth Affiliated Hospital Sun Yat‐sen University Shenzhen Guangdong 518033 China; ^4^ State Key Laboratory of Ophthalmology Zhong Shan Ophthalmic Center Sun Yat‐sen University Guangzhou Guangdong 510000 China

**Keywords:** hypogonadism, prime editing, spermatogenesis, stem Leydig cells, testis, testosterone

## Abstract

Hereditary primary hypogonadism (HPH), caused by gene mutation related to testosterone synthesis in Leydig cells, usually impairs male sexual development and spermatogenesis. Genetically corrected stem Leydig cells (SLCs) transplantation may provide a new approach for treating HPH. Here, a novel nonsense‐point‐mutation mouse model (*Lhcgr* ^W495X^) is first generated based on a gene mutation relative to HPH patients. To verify the efficacy and feasibility of SLCs transplantation in treating HPH, wild‐type SLCs are transplanted into *Lhcgr* ^W495X^ mice, in which SLCs obviously rescue HPH phenotypes. Through comparing several editing strategies, optimized PE2 protein (PEmax) system is identified as an efficient and precise approach to correct the pathogenic point mutation in *Lhcgr*. Furthermore, delivering intein‐split PEmax system via lentivirus successfully corrects the mutation in SLCs from *Lhcgr* ^W495X^ mice *ex vivo*. Gene‐corrected SLCs from *Lhcgr* ^W495X^ mice exert ability to differentiate into functional Leydig cells in vitro. Notably, the transplantation of gene‐corrected SLCs effectively regenerates Leydig cells, recovers testosterone production, restarts sexual development, rescues spermatogenesis, and produces fertile offspring in *Lhcgr* ^W495X^ mice. Altogether, these results suggest that PE‐based gene editing in SLCs ex vivo is a promising strategy for HPH therapy and is potentially leveraged to address more hereditary diseases in reproductive system.

## Graphical abstract

This study generates a novel hereditary primary hypogonadism mouse model with a nonsense‐point‐mutation in *Lhcgr* gene (*Lhcgr* ^W495X^). The optimized PE2 protein (PEmax) system is an efficient and precise approach to correct the pathogenic mutation in stem Leydig cells (SLCs) from *Lhcgr* ^W495X^ mice ex vivo. Transplantation of gene‐corrected SLCs recovers testosterone production, restarts sexual development, rescues spermatogenesis, and produces fertile offspring in *Lhcgr* ^W495X^ mice.

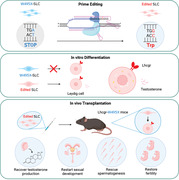

## 1 Introduction

Leydig cells (LCs), which are located in the interstitial compartment of the testes and nestled among the seminiferous tubules, are primarily responsible for the production of testosterone.^[^ ^1^ ^]^ Thus, LCs are indispensable for the development and maintenance of the masculine phenotype, endocrine homeostasis, and reproductive function.^[^ ^2^, ^3^ ^]^ Hereditary primary hypogonadism (HPH) is caused by malfunction at the level of the testes due to genetic causes which impair LCs function.^[^ ^4^ ^]^ This condition is characterized by low or absent testosterone levels and high gonadotropins levels, underdeveloped masculine phenotype, and severely impaired spermatogenesis. Luteinizing hormone/choriogonadotropin receptor (LHCGR) plays a vital role in LCs differentiation and testosterone synthesis.^[^ ^5^, ^6^ ^]^ The mutation of *Lhcgr* causes testosterone deficiency and further contributes to impaired sexual development, arrested spermatogenesis and infertility,^[^ ^7^, ^8^, ^9^ ^]^ thus serves as prototype of HPH. Testosterone replacement therapy (TRT) is the first choice for treating HPH, as exogenous TRT can largely reverse low serum levels of testosterone and partially ameliorate hypogonadism‐associated symptoms.^[^ ^10^ ^]^ However, exogenous TRT induces many adverse effects, such as sleep apnoea, stroke, heart attack, and prostate tumorigenesis.^[^ ^11^ ^]^ Notably, exogenous testosterone has yet to mimic the physiological patterns of testosterone secretion and aggravates spermatogenesis failure.^[^ ^12^ ^]^ Therefore, demands for alternative therapies to TRT are noticeably increasing.

Stem Leydig cells (SLCs), which are capable of regenerating new LCs through proliferation and differentiation, play a critical role in maintaining LCs homoeostasis in adult testes.^[^ ^13^ ^]^ SLCs from rodents have been successfully isolated, expanded and differentiated *ex vivo*, moreover, several reports demonstrated that rodents SLCs can replace senescent and chemically disrupted LCs to produce testosterone after transplantation in vivo.^[^ ^14^, ^15^ ^]^ Further experiment from our group found that autologous SLCs transplantation can increase testosterone levels, improve spermatogenesis and ameliorate relevant symptoms of primary hypogonadism in non‐human primate models.^[^ ^16^ ^]^ Recently, several groups have reported to identify human SLCs which exhibited the clonogenic, self‐renewal capacity and showed multi‐lineage differentiation potential. More importantly, human SLCs differentiated into functional LCs that produced testosterone in vitro and in vivo.^[^ ^17^, ^18^ ^]^ These studies suggest that SLCs transplantation is a promising therapy for male primary hypogonadism. However, SLCs transplantation can't be achieved due to the hereditary gene defect of SLCs from HPH patients.

In recent years, the emergence of highly versatile genome‐editing technologies has provided investigators with the ability to manipulate DNA sequences of a broad spectrum of cell types to cure hereditary diseases.^[^ ^19^, ^20^, ^21^ ^]^ For better safety profile than in vivo genome editing approaches, *ex vivo* gene editing strategies have been investigated for the prevention or treatment of hereditary disorders associated with blood, liver, islet, and skin, et al.^[^ ^22^, ^23^, ^24^, ^25^, ^26^ ^]^ Previous reports have shown that *ex vivo* gene‐modified autologous hematopoietic stem cells undergo self‐renewal and establish a population of modified cells to rescue sickle cell disease.^[^ ^22^, ^23^ ^]^ Recently, Yohan Kim and colleagues have demonstrated the therapeutic potential of *ex vivo* gene editing strategies in hepatic progenitors for the treatment of hereditary liver diseases.^[^ ^24^ ^]^ Correction of the mutation in stem cells by *ex vivo* gene editing and differentiation to pancreatic endocrine cells restored insulin production and could serve as a treatment for permanent neonatal diabetes mellitus caused by a single gene mutation.^[^ ^27^ ^]^ These large‐scale applications of gene‐edited stem cell‐derived cells in preclinical and clinical studies suggest that once hypogonadism‐inducing genetic mutations have been corrected in SLCs, they should be suitable for autologous transplantation to rescue HPH by differentiating into functional LCs.

Here, we first generated an *Lhcgr* ^W495X^ mutant mouse model based on a nonsense point mutation of *Lhcgr* that is observed in HPH patients. Next, we assessed the feasibility of SLCs transplantation therapy for HPH using SLCs from wild type mice (WT‐SLCs). We further explored editing efficiencies and off‐target effects of genome editing tools on reporter cells and mutant SLCs from *Lhcgr* ^W495X^ mice. Finally, we evaluated the therapeutic effects of *ex vivo* gene‐corrected SLCs on the HPH mouse model.

## 2 Results

### 2.1 Generation and Characterization of *Lhcgr* ^W495X^ Knock‐in Point Mutation Mice

**Figure 1:**
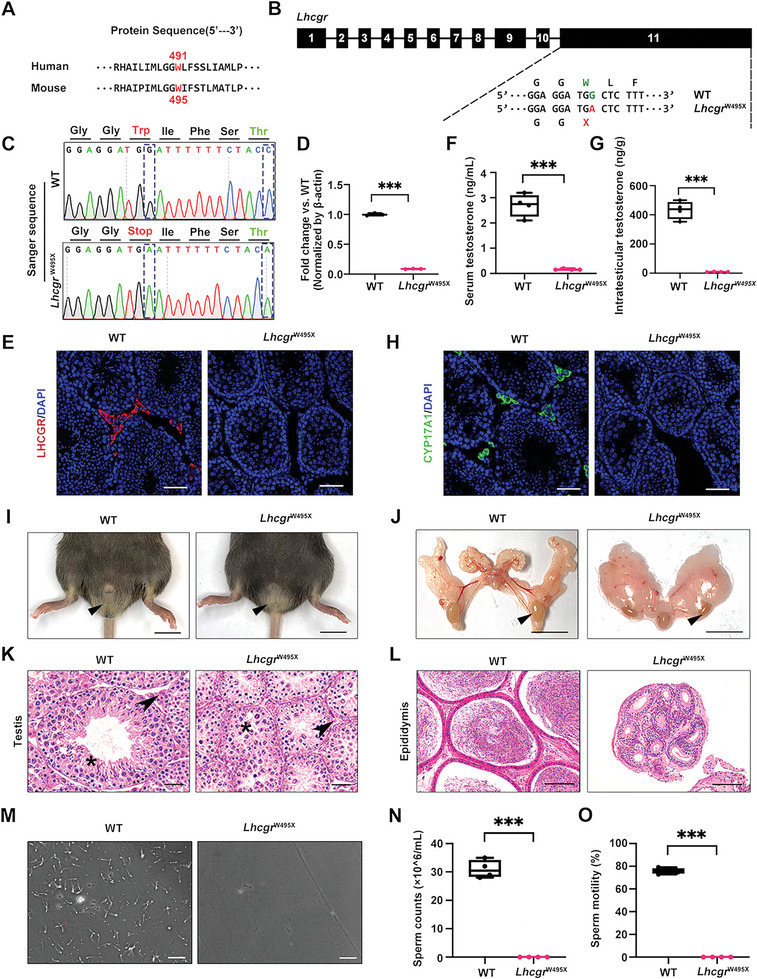
Generation and characterization of *Lhcgr* ^W495X^ knock‐in point mutation mouse. A) The alignment result of human and murine LHCGR protein sequence. B) Schematic view of the nonsense mutation at exon11 of *Lhcgr* gene in *Lhcgr* ^W495X^ mouse. C) The Sanger sequencing result of *Lhcgr* ^W495X^ (homozygote) and WT mice. D) Quantitative RT‐PCR was used to quantify *Lhcgr* mRNA transcripts in testicular tissues from 8‐week‐old WT and *Lhcgr* ^W495X^ mice (n = 3), β‐actin was used as internal control. E) The testes sections from 8‐week‐old WT and *Lhcgr* ^W495X^ mice (n = 4) were stained with LHCGR and DAPI, Scale bars: 50 µm. F) Serum testosterone concentration of 8‐week‐old WT and *Lhcgr* ^W495X^ mice (n = 4). G) Intratesticular testosterone concentration of 8‐week‐old WT and *Lhcgr* ^W495X^ mice (n = 4). H) Immunofluorescent analysis of CYP17A1 protein in testes of 8‐week‐old WT and *Lhcgr* ^W495X^ mice (n = 4). Scale bars: 50 µm. I,J) Representative photographs of external (I) and internal genitalia (J) of indicated groups (n = 4). Arrowheads indicate the scrotums (I) or testes (J) of indicated group. Scale bars: 1 cm. K) Histologic analysis of testes sections from 8‐week‐old WT and *Lhcgr* ^W495X^ mice (n = 4). Asterisk shows the seminiferous tubules and arrowhead indicates LCs. Scale bars: 25 µm. L) Representative H&E images of cauda epididymides from indicated groups (n = 4). Scale bars: 75 µm. M) Representative light micrographs of sperm obtained from 8‐week‐old WT and *Lhcgr* ^W495X^ mice (n = 4). Scale bars: 50 µm. N,O) Quantification of the sperm counts (N) and motility (O) from indicated groups. Data are represented by box plots and whiskers are minimum to maximum values. Significance was determined by two‐tailed *t*‐tests. *** P < 0.001.

We first utilized the CRISPR/Cas9 system to generate a knock‐in mouse line harboring the c.1485G>A (W495X) point mutation which mimics the c.1473G>A (W491X) mutation of *LHCGR* gene in human patients (**Figure** **1**A,B; and Figure S1A, Supporting Information).^[^ ^9^ ^]^ This mutation leads to a premature stop codon and formation of truncated, non‐functional LHCGR protein. We therefore referred homozygous W495X mutant mice as *Lhcgr* ^W495X^ and used WT littermates as an internal control for comparisons in the following experiments. Sanger sequencing results of the genomic DNA from the indicated mice validated the successful generation of *Lhcgr* ^W495X^ mice (Figure 1C). As expected, *Lhcgr* ^W495X^ mice presented an extremely low expression of *Lhcgr* mRNA in testes when compared with WT mice (Figure 1D). Immunostaining assay showed that testicular cell in *Lhcgr* ^W495X^ mice did not express LHCGR (Figure 1E). These data demonstrate the successful generation of *Lhcgr* ^W495X^ knock‐in point mutation mouse model.

The serum testosterone level of adult *Lhcgr* ^W495X^ mice was incredibly lower than that of WT littermates (Figure 1F). The concentration of intratesticular testosterone also decreased in *Lhcgr* ^W495X^ mice compared with WT mice (Figure 1G). Serum luteinizing hormone (LH) level increased in *Lhcgr* ^W495X^ mice (Figure S1B, Supporting INformation). In parallel with previous report,^[^ ^28^ ^]^ we did not observe significant changes in follicle stimulating hormone (FSH) level between two groups (Figure S1C, Supporting Information). Additionally, immunostaining assay negligibly detected LCs marker cytochrome P450 family 17 subfamily A member 1 (CYP17A1) in testes sections of *Lhcgr* ^W495X^ mice, indicating the stagnation of LCs differentiation in the mutant mice (Figure 1H). Next, we explored whether *Lhcgr* ^W495X^ mice shared the same symptom of impaired sexual development with HPH patients. Compared with WT littermates, *Lhcgr* ^W495X^ male mice had undescended and smaller testes at 8 weeks of age (Figure 1I,J). Quantitative analysis showed that the parameters of sexual development in *Lhcgr* ^W495X^ mice decreased considerably than WT mice, such as the weight of testis, epididymis, seminal vesicle, prostate, vas deferen, and the length of vas deferens, penis and anogenital distance (Figure S1D–S1K, Supporting Information).

Since infertility often occurs in HPH patients, we further evaluated spermatogenesis of 8‐week‐old male *Lhcgr* ^W495X^ mice. We found that the diameter of seminiferous tubules decreased obviously in *Lhcgr* ^W495X^ mice compared with WT mice. Moreover, elongating spermatids were absent in the seminiferous tubules of *Lhcgr* ^W495X^ mice (Figure 1K). Histological analysis of cauda epididymides demonstrated that the luminal diameters were dramatically decreased in *Lhcgr* ^W495X^ mice compared to WT mice, and epididymides lumens of *Lhcgr* ^W495X^ mice were completely devoid of sperm (Figure 1L). Further, we applied computer‐aided sperm analysis (CASA) system to determine the quantity and motility of sperm in cauda epididymides (Figure 1M). At 8‐weeks of age, normal sperm counts (31.0 ± 3.2 × 10^6^ mL^−1^) and sperm motility (75.8 ± 2.5%) were observed in WT mice, whereas sperm were not detected in *Lhcgr* ^W495X^ mice (Figure 1N,O). Quantitative RT‐PCR analysis revealed that genes specific for spermatogonia (*Dazl*, *Plzf*, and *Uchl1*) and spermatocytes (*Piwil1*, *Sycp3* and *Tex101*) were highly enriched in the testes of *Lhcgr* ^W495X^ mice (Figure S2A and S2B, Supporting Information). However, genes related to round spermatids (*Acrv1*, *Tsga8*, and *Tssk1*) and elongating spermatids (*Asb9*, *Tnp2*, and *Prm2*) showed obvious decline in the testes of *Lhcgr* ^W495X^ mice compared with WT mice (Figure S2C and S2D, Supporting Information). These results suggested that spermatogenesis was arrested at the stage of round spermatids in adult *Lhcgr* ^W495X^ mice. Consistently, the acrosome marker peanut agglutinin (PNA) showed incredibly weak signals and the elongating spermatids marker transition protein 2 (TNP2) showed an absent signal in the testes of *Lhcgr* ^W495X^ mice (Figure S2E–S2J, Supporting Information). These data demonstrate that the *Lhcgr* ^W495X^ mice mimics the pathological and clinical features of human HPH, and thus serve as an ideal model for the research of HPH.

### 2.2 WT‐SLCs Transplantation Recovers Sex Hormone Level and Restarts Sexual Development in *Lhcgr* ^W495X^ Mice

To investigate the potential of SLCs‐based therapy for HPH, we first evaluated the feasibility of WT‐SLCs transplantation in *Lhcgr* ^W495X^ mice. As previously reported,^[^ ^29^ ^]^ we sorted CD51^+^ SLCs from the testes of 7‐day‐old WT mice by fluorescence activated cell sorting (FACS) (Figure S3A and S3B, Supporting Information). These freshly isolated CD51^+^ SLCs were then cultured on plastic dishes in expansion medium at a density of 2 × 10^5^ cells mL^−1^. One day later, the attached SLCs presented a typically spindle shape (Figure S3C, Supporting Information), moreover, 97.1 ± 1.4% and 95.2 ± 2.7% of them expressed SLCs markers Nestin and platelet‐derived growth factor receptor alpha (PDGFRα) respectively (Figure S3D–S3F, Supporting Information). To trace the transplanted cells in vivo, we transduced WT‐SLCs with lentiviruses expressing mCherry under the control of the CAG promoter (Figure S3G, Supporting Information). The transfected cells stably expressed mCherry were sorted by FACS and used in the following experiments (Figure S3H–S3J, Supporting Information).

**Figure 2:**
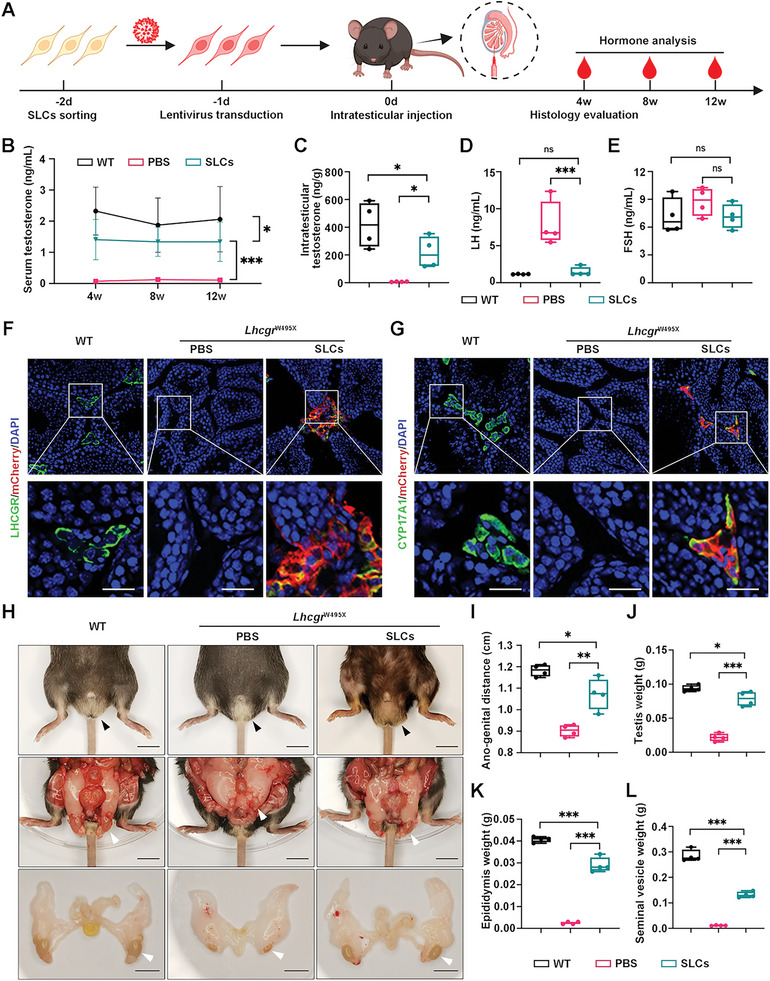
WT‐SLCs transplantation restarts sexual development and recovers sex hormone level in *Lhcgr* ^W495X^ mice. A) Experiment overview of the in vivo studies. B) Serum testosterone concentration of WT and *Lhcgr* ^W495X^ mice injected with PBS or WT‐SLCs (8 × 10^4 cells/testis) at 4 weeks, 8 weeks, 12 weeks after treatment (n = 4). C–E) Intratesticular testosterone (C), LH (D), and FSH (E) level of the indicated groups at 4 weeks after treatment (n = 4). F,G) Representative confocal images of testes sections from indicated groups. The sections were stained with LCs markers (LHCGR and CYP17A1), mCherry, and DAPI. mCherry indicates transplanted SLCs. Scale bars: 25 µm. H) Representative images of external and internal genitalia of WT and *Lhcgr* ^W495X^ mice injected with PBS or WT‐SLCs (8×10^4 cells/testis) at 4 weeks after treatment (n = 4). Arrowheads indicate the scrotums or testes of indicated groups. Scale bars: 1 cm. I–L) The length of ano‐genital distance (I) and the weight of testis (J), epididymis (K), seminal vesicle (L) of indicated group (n = 4). Data are represented by mean ± SD (B) or box plots with minimum to maximum values (C,D,E,I,J,K,L). Significance was determined by two‐way repeated measures ANOVA (B) or one‐way ANOVA (C‐E,I‐L). * *p* < 0.05, ** *p* < 0.01, *** *p* < 0.001, ns = not significant.

Next, the mCherry^+^ SLCs were transplanted into the testes of 8‐week‐old *Lhcgr* ^W495X^ mice. WT mice were used as positive control, while the *Lhcgr* ^W495X^ mice receiving intratesticular PBS injection were regarded as negative control (**Figure** **2**A). Four weeks after WT‐SLCs transplantation, the serum testosterone level of *Lhcgr* ^W495X^ mice increased to about 60.5% of that in WT mice. In contrast, serum testosterone level showed no increase in PBS‐treated mice. We also evaluated serum testosterone for a longer period of time and observed that testosterone levels maintained stable at 8 weeks and 12 weeks after SLCs transplantation (Figure 2B). Quantification of intratesticular testosterone also revealed a significant increase in WT‐SLCs transplantation group (217.9 ± 114.2 ng g^−1^) compared with PBS‐treated group (7.0 ± 1.6 ng g^−1^) (Figure 2C). As expected, LH levels decreased significantly 4 weeks after SLCs transplantation compared to PBS injection (Figure 2D). We did not detect significant differences in FSH levels among the three groups (Figure 2E). These results reflect the normal negative feedback of hypothalamic‐pituitary‐gonad axis to increased testosterone level after SLCs transplantation. Additionally, immunofluorescent analysis revealed that mCherry‐labelled cells co‐expressed LHCGR and CYP17A1 in testicular interstitium of WT‐SLCs transplantation group, while there was no positive signal of LHCGR and CYP17A1 in PBS‐treated group (Figure 2F,G). In addition, 15.6 ± 7.1% of the mCherry^+^ cells were positive for the pan‐cell‐cycle marker Ki67 (Figure S4, Supporting Information), indicating the measurable proliferative activity of the transplanted SLCs.

Intriguingly, we found that the retained testes descended into the scrotum in *Lhcgr* ^W495X^ mice at 4 weeks after WT‐SLCs transplantation. Moreover, the underdeveloped external genitals (penis and scrotum) of the WT‐SLCs‐treated *Lhcgr* ^W495X^ mice approached the size of those observed in WT mice (Figure 2H). We also observed normalization of sexual development in *Lhcgr* ^W495X^ mice after WT‐SLCs transplantation (Figure 2I–L; Figure S5, Supporting Information). Overall, these data demonstrate that transplanted SLCs give rise to LCs which produce testosterone in vivo and restart sexual development.

### 2.3 WT‐SLCs Transplantation Rescues Spermatogenesis in *Lhcgr* ^W495X^ Mice

**Figure 3:**
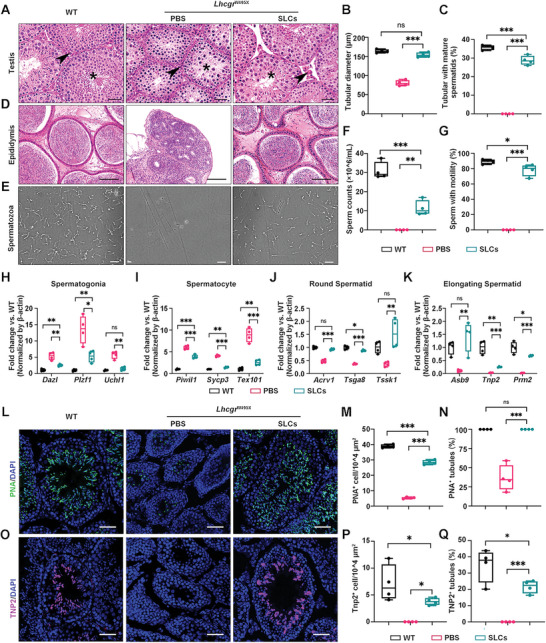
WT‐SLCs transplantation promotes spermatogenesis in *Lhcgr* ^W495X^ mice. A) Representative H&E staining images of testes sections obtained from WT and *Lhcgr* ^W495X^ mice injected with PBS or WT‐SLCs (8 × 10^4 cells/testis) (n = 4). Asterisk shows the seminiferous tubules and arrowhead indicates LCs. Scale bars: 25 µm. B) Seminiferous tubule diameter of indicated groups, a total of random 400 seminiferous tubules from 4 independent samples were calculated per group. C) The proportion of seminiferous tubules with mature spermatozoa in indicated groups (n = 4). D) Histology analysis of cauda epididymides of indicated groups (n = 4). Scale bars: 75 µm. E) Representative light micrographs of sperm acquired from indicated groups (n = 4). Scale bars: 50 µm. F,G) Sperm counts (F) and motility (G) of indicated groups (n = 4). H–K) Quantitative RT‐PCR analysis of spermatogonia (*Dazl*, *Plzf*, and *Uchl1*, H), spermatocyte (*Piwil1*, *Sycp3*, and *Tex101*, I), round spermatid (*Acrv1*, *Tsga8*, and *Tssk1*, J), and elongating spermatid (*Asb9*, *Tnp2*, and *Prm2*, K) of indicated groups (n = 4), β‐actin was used as internal control. L–N) Immunofluorescence staining of acrosome marker PNA (L) in testes sections, and quantitative analysis the number of PNA^+^ cells in 10^4 µm^2^ area (M) and the percentage of PNA^+^ tubules (N) from indicated groups (n = 4). Three sections per slide and three slides per testis were randomly selected and evaluated. Scale bars: 50 µm. O–Q) Immunofluorescence staining of elongating spermatid marker TNP2 (O) in testes sections, and quantitative analysis the number of TNP^+^ cells in 10^4 µm^2^ area (P) and the percentage of TNP2^+^ tubules (Q) from indicated groups (n = 4). Three sections per slide and three slides per testis were randomly selected and evaluated. Scale bars: 50 µm. Data are represented by box plots, and whiskers are minimum to maximum values. Significance was determined by one‐way ANOVA. * *p* < 0.05, ** *p* < 0.01, *** *p* < 0.001, ns = not significant.

Given the condition that WT‐SLCs transplantation could recover LCs function and restart sexual development, we next surveyed whether SLCs transplantation could rescue spermatogenesis in *Lhcgr* ^W495X^ mice. Four weeks after transplantation, the seminiferous tubule diameter increased in WT‐SLCs‐treated group (154.0 ± 6.3 µm) compared with PBS‐treated group (80.2 ± 7.5 µm), approaching the levels observed in WT mice (164.0 ± 4.7 µm) (**Figure** **3**A,B). When calculating the percentage of seminiferous tubules that contained mature sperms, we found that ≈28.7% of the tubules in *Lhcgr* ^W495X^ mice injected with WT‐SLCs contained mature sperms, whereas spermatogenesis remained arrested without any sperm in PBS‐treated *Lhcgr* ^W495X^ mice (Figure 3C).

To further evaluate spermatogenesis after WT‐SLCs transplantation, epididymides samples were collected from the three groups at 4 weeks after treatment. Histological analysis showed that treatment of *Lhcgr* ^W495X^ mice with WT‐SLCs restored the luminal diameter in the cauda epididymides with the presence of massive sperms at this location (Figure 3D,E). Consistently, epididymal sperm number of the WT‐SLCs transplantation group was increased to over one third of that observed in WT mice, whereas sperm were not detected in PBS‐treated *Lhcgr* ^W495X^ mice (Figure 3F). Moreover, sperm motility of *Lhcgr* ^W495X^ mice increased markedly under WT‐SLCs transplantation, almost reaching the level observed in WT mice (Figure 3G).

Further, the recovery of spermatogenesis was molecularly characterized by quantitative RT‐PCR for functionally defined genes reflecting spermatogonia, spermatocytes, round spermatids, and elongating spermatids. As expected, the expression of spermatogonia and spermatocytes related genes decrease relatively while round and elongating spermatids related genes increased dramatically in WT‐SLCs treated testes compared with PBS‐treated testes (Figure 3H–K). The recovery of spermatogenesis was also determined by immunostaining for PNA and TNP2 which were greatly upregulated in the testes of WT‐SLCs treated group compared with PBS‐treated *Lhcgr* ^W495X^ mice (Figure 3L–Q). Collectively, these results indicate that SLCs transplantation rescues spermatogenesis in the mouse model of HPH.

### 2.4 Prime Editors Work Better Than Adenine Base Editors in Correcting *Lhcgr* ^W495X^

**Figure 4:**
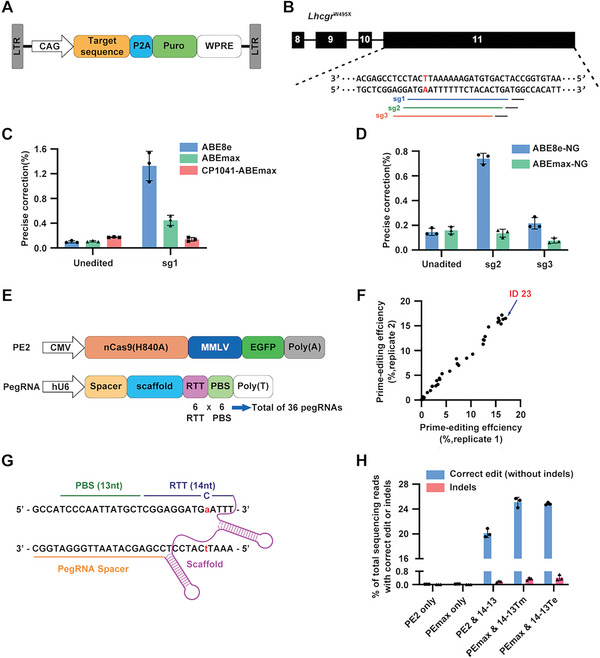
Correcting the *Lhcgr* mutation by adenine base editors and prime editors using target sequence‐containing HEK293T reporter cells. A) Schematic of the lentiviral vector containing target sequence used to generate reporter cell line. B) Diagram of sgRNAs based on ABEs to correct the point mutation (shown in red) in *Lhcgr*. The protospacer of sg1, sg2, and sg3 are represented by blue, green, and brown lines respectively. PAM of each target sequence is indicated with black lines. C) Precise correction rates of three ABEs analyzed by HTS in HEK293T reporter cells. Any read containing bystander edits was not counted. D) Precise correction rates of two ABE‐NG variants analyzed by HTS. Any read containing bystander edits was not counted. E) Schematic of PE2‐encoding plasmid and pegRNA‐encoding plasmid. Total of 36 pegRNAs‐encoding plasmids containing 6 RTTs (11 to 16 nt) and 6 PBSs (9, 10, 11, 12, 13, and 15 nt) were constructed. F) HTS outcome of each pegRNA. The editing efficiency was normalized by subtracting the average edit frequencies in the control group (only transfected with PE2‐encoding plasmid). The ID of pegRNA that achieved highest editing efficiency is indicated with an arrow and red highlights. G) Schematic view of peg14‐13 (pegRNA with 14 nt RTT and 13 nt PBS), which achieved highest correction rate in pegRNA screening. H) The editing efficiencies of PE2 combining with peg14‐13 and PEmax combining with epegRNAs. Indels are plotted as red column. Data are represented by mean ± SD of independent transfections (n = 3).

On the basis of the above favorable results, we sought to establish a precise gene correction strategy for SLCs from *Lhcgr* ^W495X^ mice (W495X‐SLCs) to determine the therapeutic potential of patient‐specific and gene corrected SLCs transplantation. To evaluate the editing efficiencies of different tools at the mutant site, we generated a HEK293T reporter cell line with stably integrated *Lhcgr* ^W495X^ mutation site through lentivirus (referred to as W495X‐293) (**Figure** **4**A). First, adenine base editors (ABEs) were applied to convert the mutant “A” to the normal “G” in W495X‐293 reporter cells. We first designed a single‐guide RNA (sgRNA, sg1) containing an “NGG” protospacer adjacent motif (PAM) targeted the mutant “A” (Figure 4B). Three types of ABEs‐encoding (ABE8e, ABEmax, CP1041‐ABEmax) plasmids combining with sg1 RNA were transfected to correct the pathogenic point mutation in W495X‐293 respectively.^[^ ^30^, ^31^, ^32^ ^]^ 72 h after transfection, high‐throughput sequencing (HTS) results showed that ABE8e induced the highest precise correction (1.33 ± 0.24%) of the pathogenic mutation (A4) whereas a bystander (A5) was more efficiently converted (34.52 ± 1.26%) (Figure 4C; Figure S6A, Supporting Information). To reduce bystander edits, we tested two more sgRNAs (sg2, sg3) with two ABE variants (ABEmax‐NG, ABE8e‐NG) which could recognize relaxed PAMs.^[^ ^32^, ^33^ ^]^ HTS analysis revealed that ABE8e‐NG combined with sg2 induced the highest editing efficiency, but the mutant “A” was rarely corrected (0.74 ± 0.04%) (Figure 4D; Figure S6A, Supporting Information). Together, our results shown that these established ABE‐based strategies were less effective in correcting *Lhcgr* ^W495X^.

Next, we tested whether prime editor could effectively correct the point mutation of *Lhcgr* ^W495X^. Prime editor 2 (PE2) system requires a prime‐editing guide RNA (pegRNA) which contains a spacer sequence, a reverse transcription template (RTT) and a primer binding site (PBS).^[^ ^21^ ^]^ The target sequence for PE2 was decided by evaluating the DeepSpCas9 score on the range of −40 base pairs (bp) to +40 bp from the mutation site (counting the mutation as position 0).^[^ ^34^ ^]^ To optimize PE2 editing efficiency, we designed various pegRNAs containing 6 RTT (11 to 16 nt) and 6 PBSs (9 to 15 nt, except 14 nt, because it ends with “T”) of different lengths (Figure 4E). Subsequently, we co‐transfected PE2‐encoding plasmids with 36 pegRNA‐encoding plasmids into W495X‐293 respectively. Ultimately, we selected pegRNA ID 23 (named as peg14‐13) which resulted in the highest correction rate (16.75 ± 0.35%) in W495X‐293 (Figure 4F,G). Previous studies had shown that PE3 system (PE2 plus an additional nicking sgRNA) may produce higher editing efficiency than PE2,^[^ ^35^ ^]^ thus we evaluated editing efficiency of PE3 in W495X‐293. Unfortunately, three nicking sgRNAs did not significantly increase editing efficiency in this point mutation (Figure S6B and S6C, Supporting Information). Recently, engineered pegRNAs (epegRNA) and optimized PE2 protein (PEmax) were reported to be conducive to improve prime editing efficiency.^[^ ^36^, ^37^ ^]^ We constructed a trimmed evopreQ epeg14‐13 (named as epeg14‐13Te) and a trimmed mpknot epeg14‐13 (named as epeg14‐13Tm), and then test them in W495X‐293. HTS results showed that PEmax synergizing with peg14‐13Tm achieved the highest editing efficiency (25.1 ± 0.79%) with low rates of indels (0.34 ± 0.08%) (Figure 4H; Figure S6D, Supporting Information). In total, we compared various mutation correction strategies based on ABEs and PEs in W495X‐293 and identified PEmax along with epeg14‐13Tm was the best choice in correction of *Lhcgr* ^W495X^.

### 2.5 Prime Editing Corrects Mutant *Lhcgr* in W495X‐SLCs

**Figure 5:**
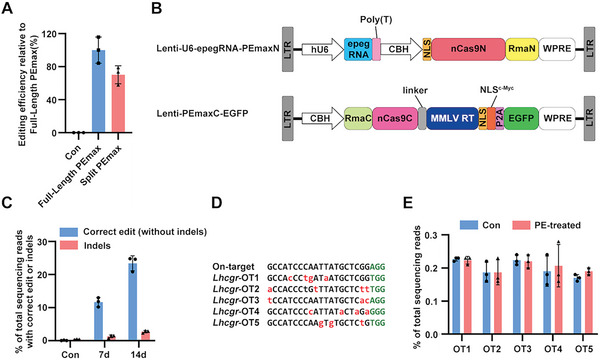
Editing efficiency and off‐target effects of PEmax system in W495X‐SLCs. A) Editing efficiency of control, Full‐Length PEmax and Split PEmax. B) Schematic view of dual lentiviral vectors encoding intein‐split PEmax and epegRNA. C) Precise correction (blue column) and indels rates (red column) in edited W495X‐SLCs analyzed by HTS at 7 and 14 days after treatment with PEmax system. D) Potential off‐target sites predicted by CRISPOR. Mismatch nucleotides were indicated in red; PAM was indicated in green. E) Off‐target analysis in edited W495X‐SLCs 14 days after treatment with PEmax system. Data are represented by mean ± SD of independent transfections (n = 3).

We next assessed the editing efficiency of this PEmax system in W495X‐SLCs. Considering the packaging capacity limit of lentivirus, we here introduced the reported 1024–1025 splitting site^[^ ^38^ ^]^ into PEmax system and tested whether split‐PEmax also works in W495X‐293 reporter cells (Figure S7A, Supporting Information). 72 h after transfection, we analyzed sanger sequencing data using BEAT program.^[^ ^39^ ^]^ Compared to the full‐length PEmax, split‐PEmax retained about 70% activity for “A” to “G” conversion of *Lhcgr* ^W495X^ nonsense mutation (**Figure** **5**A), suggesting split‐PEmax could be used in W495X‐SLCs with acceptable editing efficiency.

Next, we isolated CD51^+^ W495X‐SLCs from 7‐day‐old *Lhcgr* ^W495X^ mice by FACS and identified that over 97% of these cells express Nestin and PDGFRα (Figure S7B–S7G, Supporting Information). Thereafter, we transduced N‐terminal half (PEmaxN, containing epegRNA) and C‐terminal half (PEmaxC, containing EGFP) lentivirus into W495X‐SLCs and observed an average correction rate of 11.67% at 7 days and 23.42% at 14 days (Figure 5B,C; Figure S7H and S7I, Supporting Information). We also detected low rates of indels (2.73 ± 0.36%) 14 days after transduction (Figure 5C; Figure S8, Supporting Information). To assess off‐target editing induced by PE, we chose five potential off‐target sites predicted by CRISPOR (Figure 5D).^[^ ^40^ ^]^ We performed targeted deep sequencing at these predicted sites and didn't observe any detectable off‐target edits compared with the control (only transduced with PEmaxC lentivirus) (Figure 5E). Overall, these results suggest that PEmax could induce precise and specific mutation correction in W495X‐SLCs.

### 2.6 PEmax‐Edited W495X‐SLCs Differentiate into LCs In Vitro

**Figure 6:**
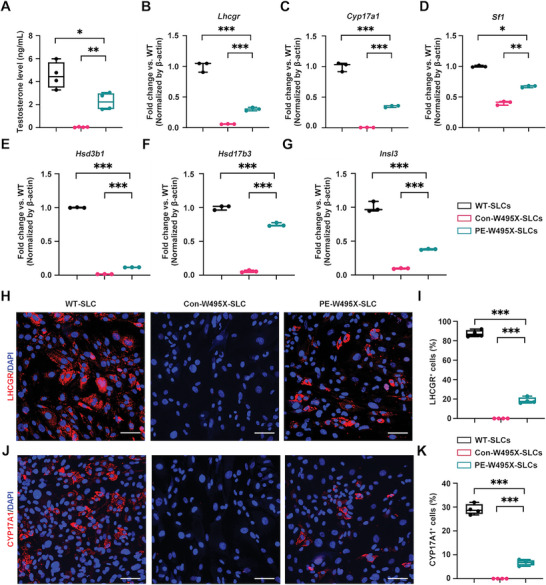
PEmax‐edited W495X‐SLCs differentiate into LCs in vitro. A) The testosterone concentration in the supernatant of WT‐SLCs, Con‐W495X‐SLCs, and PE‐W495X‐SLCs groups 14 days after differentiation in vitro (n = 4). B–H) Quantitative RT‐PCR analysis of LCs markers *Lhcgr* (B), *Cyp17a1* (C), *Sf1* (D), *Hsd3b1* (E), *Hsd17β3* (F) and *Insl3* (G) of induced SLCs in indicated groups (n = 3). β‐actin was used as internal control. H–K) Immunofluorescence staining of LCs markers LHCGR (H) and CYP17A1 (J) of indicated SLCs 14 days after differentiation in vitro (n = 3). The nuclei were counter‐stained with DAPI. Scale bars: 75 µm. Quantitative analysis the proportion of LHCGR (I) and CYP17A1 (K) positive cells of indicated groups (n = 4). Six regions per slide were randomly selected and evaluated. Data are represented by box plots, and whiskers are minimum to maximum values. Significance was determined one‐way ANOVA. * *p* < 0.05, ** *p* < 0.01, *** *p* < 0.001.

We next evaluated whether the PEmax‐edited W495X‐SLCs (PE‐W495X‐SLCs) had normal function in differentiating into LCs and then produce testosterone in vitro. After 14 days of culture with differentiation‐inducing medium (DIM), the testosterone level in supernatant of PE‐W495X‐SLCs group increased strikingly at an average of 2.28 ng mL^−1^, compared with 0.04 ng mL^−1^ of control W495X‐SLCs group which only received PEmaxC lentivirus transduction (Con‐W495X‐SLCs) (**Figure** **6**A). As expected, quantitative RT‐PCR revealed that the expression of *Lhcgr* increased considerably in PE‐W495X‐SLCs group compared with Con‐W495X‐SLCs group (Figure 6B). Additionally, the transcript levels of LCs markers *Cyp17a1, Sf1, Hsd3b1, Hsd17b3*, and *Insl3* were significantly higher in PE‐W495X‐SLCs group than Con‐W495X‐SLCs group (Figure 6C–G). The results were further verified by the immunofluorescence analysis. 87.4 ± 3.5% and 29.0 ± 2.2% of WT‐SLCs expressed LHCGR and CYP17A1 at 14 days after induction (Figure 6H–K). After the mutant point was repaired through prime editing, 18.5 ± 3.1% and 6.7 ± 1.2% of induced cells were stained with LHCGR and CYP17A1 respectively, however, there was no cell expressing LHCGR and CYP17A1 in Con‐W495X‐SLCs group (Figure 6H–K). These data suggest that gene‐corrected SLCs from *Lhcgr* ^W495X^ mice had ability in differentiating into functional LCs in vitro.

### 2.7 Transplantation of PEmax‐Edited W495X‐SLCs into *Lhcgr* ^W495X^ Mice Rescues HPH Phenotypes In Vivo

**Figure 7:**
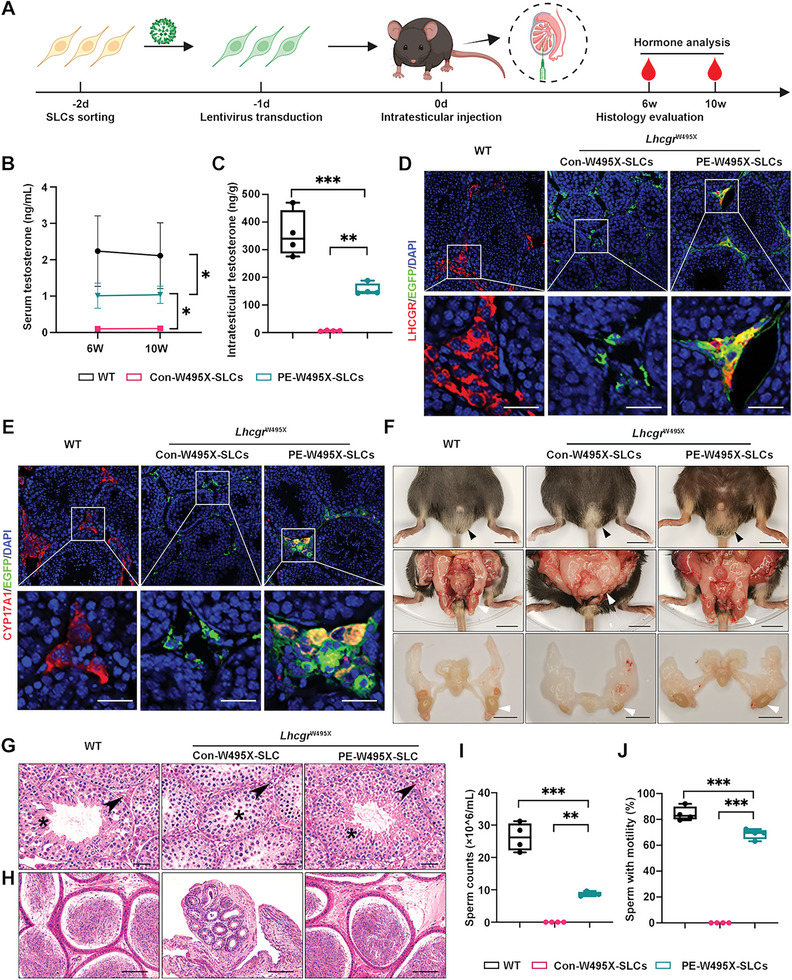
PEmax‐edited W495X‐SLCs transplantation rescues HPH phenotypes in *Lhcgr* ^W495X^ mice. A) Experiment overview of the in vivo studies. B) Serum testosterone concentration of indicated groups at 6 weeks and 10 weeks after treatment (n = 4). C) Intratesticular testosterone concentration in *Lhcgr* ^W495X^ mice at 6 weeks after transplantation (n = 4). D,E) Representative confocal images of testes sections from *Lhcgr* ^W495X^ mice at 6 weeks after transplantation. The sections were stained with LCs markers (LHCGR and CYP17A1), EGFP, and DAPI. EGFP indicates transplanted SLCs. Scale bars: 25 µm. F) Representative images of genitalia of WT, and *Lhcgr* ^W495X^ mice injected with Con‐W495X‐SLCs (4 × 10^5 cells testis^−1^) or PE‐W495X‐SLCs (4 × 10^5 cells testis^−1^) at 6 weeks after treatment (n = 4). Arrowheads indicate the scrotums or testes of indicated groups. Scale bars: 1 cm. G) Histological analysis of testes sections from WT and *Lhcgr* ^W495X^ mice at 6 weeks after transplantation (n = 4). Asterisk shows the seminiferous tubules and arrowhead indicates LCs. Scale bars: 25 µm. H) Histological analysis of cauda epididymides sections from WT and *Lhcgr* ^W495X^ mice at 6 weeks after transplantation (n = 4). Scale bars: 75 µm. I,J) Quantitative analysis of sperm counts (I) and motility (J) in cauda epididymides from *Lhcgr* ^W495X^ mice at 6 weeks after transplantation (n = 4). Data are represented by mean ± SD (B) or box plots with minimum to maximum values (C,I,J). Significance was determined by two‐way repeated measures ANOVA (B) or one‐way ANOVA (C,I,J). * *p* < 0.05, ** *p* < 0.01, *** *p* < 0.001.

To explore the therapeutic potential of gene‐corrected W495X‐SLCs in vivo, we next transplanted PE‐W495X‐SLCs into the testes of 8‐week‐old *Lhcgr* ^W495X^ mice. WT mice and *Lhcgr* ^W495X^ mice receiving Con‐W495X‐SLCs injection were used as control groups. Considering PEmax system needed about 2 weeks to reach certain editing efficiency in W495X‐SLCs, the therapeutic effects were analyzed at 6 weeks and 10 weeks after transplantation (**Figure** **7**A). Similar to the results obtained in WT‐SLCs transplantation cohort, *Lhcgr* ^W495X^ mice showed a considerable recovery of serum testosterone concentration, accounting for ≈45% of WT mice at 6 weeks after PE‐W495X‐SLCs transplantation. Moreover, treated mice in PE‐W495X‐SLCs group maintained a stable testosterone concentration until 10 weeks after transplantation (Figure 7B). The intratesticular testosterone level of *Lhcgr* ^W495X^ mice was also recovered at 6 weeks after PE‐W495X‐SLCs treatment (Figure 7C). Immunofluorescence analysis revealed that the transplanted PE‐W495X‐SLCs could give rise to LCs in vivo, as evidenced by the expression of LHCGR and CYP17A1 (Figure 7D,E). Moreover, 9.1 ± 5.6% of the PE‐W495X‐SLCs were positive for the proliferation marker Ki67 (Figure S9, Supporting Information). Additionally, we observed normalization of sexual development in *Lhcgr* ^W495X^ mice after PE‐W495X‐SLCs transplantation (Figure 7F). PE‐W495X‐SLCs transplantation achieved significant recovery of spermatogenesis as inferred by the appearance of elongating spermatids in testes (Figure 7G), the presence of sperm in the caudal epididymides (Figure 7H) and significantly increased semen parameters (Figure 7I,J) from *Lhcgr* ^W495X^ mice. To sum up, PEmax‐edited W495X‐SLCs had normal function in vivo and could rescue HPH phenotypes in *Lhcgr* ^W495X^ mice.

### 2.8 Transplantation of PEmax‐Edited W495X‐SLCs into *Lhcgr* ^W495X^ Mice Restores Fertility and Produces Fertile Offspring

**Figure 8:**
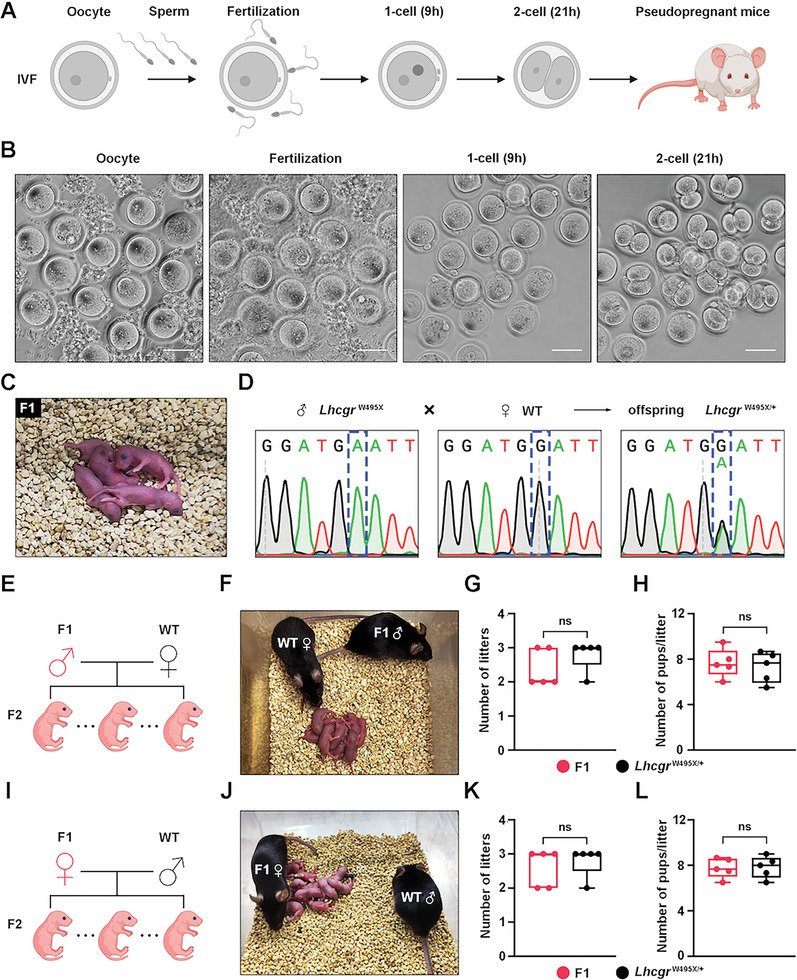
PEmax‐edited W495X‐SLCs transplantation restores fertility and produces fertile offspring. A) The in vitro fertilization (IVF) scheme used to produce offspring with sperm from PEmax‐edited W495X‐SLCs‐treated *Lhcgr* ^W495X^ mice (n = 3) and oocytes from WT females. B) Representative images of oocytes, fertilization, 1‐cell embryo, and 2‐cell embryo. Scale bars: 50 µm. C) The offspring (F1) derived from PE‐W495X‐SLCs‐treated *Lhcgr* ^W495X^ male mice. D) The Sanger sequencing result of the pups derived from PE‐W495X‐SLCs‐treated *Lhcgr* ^W495X^ males and WT females. E) Mating scheme used to produce the second generation (F2) mice. F) The male F1 mice were used to produce F2 by mating with WT females. G,H) Continuous breeding assay starting at 8 weeks of age, showing numbers of litters (G) and pups per litter (H) between F1 males (n = 5) and *Lhcgr* ^W495X/+^ males (n = 5) within 3 months. I) Mating scheme used to produce F2 mice. J) The female F1 mice were used to produce F2 pups by mating with WT males. K,L) Continuous breeding assay starting at 8 weeks of age, showing numbers of litters (K) and pups per litter (L) between F1 females and *Lhcgr* ^W495X/+^ females within 3 months (n = 5). Data are represented by box plots, and whiskers are minimum to maximum values. Significance was determined by Mann‐Whitney U tests (G,K) two‐tailed *t*‐test (H,L). ns = not significant.

To assess whether the sperm produced after PE‐W495X‐SLCs transplantation could support reproduction, in vitro fertilization (IVF) was performed using spermatozoa obtained from the caudal epididymides of male *Lhcgr* ^W495X^ mice at 6 weeks after PE‐W495X‐SLCs injection and oocytes harvested from female WT mice (**Figure** **8**A). Among a total of 489 eggs used for IVF, 191 (39.1%) successfully progressed to 2‐cell embryos in vitro (efficiency, 37.7% to 42.2%). Of these 2‐cell embryos, 98 were transplanted into the oviducts of 5 pseudo‐pregnant mice, which produced 36 offspring (efficiency 25% to 45%) (Figure 8B,C; Table S1, Supporting Information). To confirm that the offspring were derived from the PE‐W495X‐SLCs‐treated *Lhcgr* ^W495X^ male mice and WT females, tail DNA from the ten representative pups were subjected to perform PCR and Sanger sequencing. The results showed that the ten pups carried the WT and mutated alleles at proportions consistent with Mendelian law (Figure 8D).

We next investigated whether the offspring generated via PE‐W495X‐SLCs transplantation could produce a second generation by natural mating. Five adult males and five females generated from PE‐W495X‐SLCs‐treated *Lhcgr* ^W495X^ mice were mated with corresponding WT mice, and all were proven fertile by natural mating (Figure 8E–L). Moreover, the offspring from PE‐W495X‐SLCs‐treated *Lhcgr* ^W495X^ mice exhibited normal fertility compared to that of heterozygous mice (*Lhcgr* ^W495X/+^) (Figure 8E–L). Collectively, PEmax‐edited W495X‐SLCs treatment in *Lhcgr* ^W495X^ mice gives rise to fertile offspring.

## 3 Discussion

We herein report the first proof‐of‐concept study demonstrating that PEmax mediated gene correction in mutant SLCs *ex vivo* recovers testosterone production, restarts sexual development, rescues spermatogenesis, and restores fertility in the *Lhcgr* ^W495X^ mouse model of HPH, which provide a basis for the treatment of hereditary diseases in reproductive system.

The main clinical manifestations of HPH are testosterone deficiency, underdeveloped sexual organs, and spermatogenesis failure, which seriously affect not only men's reproductive functions, but also their overall health status and quality of life.^[^ ^4^, ^41^ ^]^ Recently, we reported that AAV‐mediated *Lhcgr* overexpression could promote LCs maturation and rescue HPH phenotype in an *Lhcgr* knockout mouse model, suggesting that the AAV‐mediated gene therapy appears to be a promising treatment for HPH.^[^ ^42^ ^]^ However, the clinical application of this strategy may involve potential challenges such as safety concern associated with viral delivery into testes. Previous studies have demonstrated that SLCs are capable of regenerating functional LCs and show great promise for the treatment of primary hypogonadism in rodents and non‐human primates.^[^ ^15^, ^16^, ^43^ ^]^ To explore the feasibility of SLCs transplantation in treating HPH, we injected WT‐SLCs into the testes of *Lhcgr* ^W495X^ mice and observed an obvious recovery of testosterone production, sexual development, and spermatogenesis after treatment. Notably, serum testosterone of *Lhcgr* ^W495X^ mice receiving WT‐SLCs transplantation maintained at least 12 weeks, suggesting that SLCs transplantation may ensure long‐term benefits by a single treatment. Consequently, SLCs transplantation may represent a safe strategy with long‐term therapeutic benefits for HPH.

Though ex vivo gene editing of stem cells has been successfully applied to treat various genetic diseases in animal models and humans,^[^ ^24^, ^44^, ^45^ ^]^ there have relatively few studies applying the strategy in the testis, possibly due to ethical considerations. As reported recently, several groups have attempted to conduct gene editing on spermatogonia stem cells (SSCs).^[^ ^46^, ^47^, ^48^ ^]^ For example, Li et al. reported that the pathogenic mutation in SSCs could be corrected *ex vivo* using CRISPR/Cas9.^[^ ^46^ ^]^ Furthermore, transplantation of gene‐corrected SSCs restored spermatogenesis of *Kit* ^w^/*Kit* ^wv^ mouse with nonobstructive azoospermia.^[^ ^46^ ^]^ However, gene correction targeting the somatic cell of male reproductive system has yet to be reported. In the present study, we isolated mutant SLCs from testis of *Lhcgr* ^W495X^ mouse model and corrected the pathogenic point mutation using prime editor. After transplantation of gene‐corrected SLCs, we observed considerable LCs maturation and testosterone recovery, obvious improvement of sexual development in *Lhcgr* ^W495X^ mice. More importantly, PE‐W495X‐SLCs‐treated *Lhcgr* ^W495X^ mice produced fertilization‐competent spermatozoa, and effectively gave rise to offspring. Notably, the offspring from PE‐W495X‐SLCs‐treated *Lhcgr* ^W495X^ mice produced the second generation by natural mating and exhibited normal fertility. To our knowledge, this is the first study to correct pathogenic mutation of somatic cells using gene editing tools in reproductive system, which is expected to be devoid of ethical issues. Moreover, current techniques allow propagation of human SLCs ex vivo,^[^ ^16^ ^]^ making it hopeful to obtain large scale of gene‐corrected cells and successful autologous transplantation in future clinical applications.

Previous reports have shown that functional *LHCGR* in human is compulsory for fetal Leydig cells (FLCs) development and their androgen synthesis.^[^ ^6^ ^]^ Thus, humans with completely inactivating *LHCGR* mutation are lacking masculinization in utero.^[^ ^49^ ^]^ However, FLCs are regulated by LH, corticotrophin‐releasing hormone (CRH), and adrenocorticotropic hormone (ACTH), raising the possibility that these hormones may act, in a redundant fashion, to stimulate fetal androgen production and induce masculinization in mice.^[^ ^50^ ^]^ Consistently, the W495X mutation in *Lhcgr* does not affect either quantity or development of FLCs. Moreover, male *Lhcgr* ^W495X^ mice were normally masculinized at birth (data not shown). Despite these differences, the results in the present study still provide a possibility of translational potential for HPH treatment in humans. In accordance with the results in mouse experiments, numerous clinical studies have identified *LHCGR* mutations which have mild damage of fetal testis development but hinder LCs development postnatally.^[^ ^51^, ^52^ ^]^ We therefore speculate that gene‐corrected human SLCs may provide ideal treatment for HPH caused by *LHCGR* mutation. Additionally, the extensive generation and study of mouse mutants and molecular analysis of human patients have revealed a number of candidate genes that are involved in HPH, including 3β‐hydroxysteroid dehydrogenase type 2 (*3β‐HSD2*),^[^ ^53^ ^]^ cytochrome P450 oxidoreductase (*POR*),^[^ ^54^ ^]^ *CYP17A1*,^[^ ^55^ ^]^ or 17β‐hydroxysteroid dehydrogenase type 3 (*17β‐HSD3*).^[^ ^56^ ^]^ Based on the therapeutic effects of gene‐corrected SLC on *Lhcgr*‐mutant HPH mice, it is rational to hypothesize that genetically manipulating SLCs exhibit strong potential to exert favorable effects on other types of genetic HPH.^[^ ^57^ ^]^ We recognize that *LHCGR* gene and FLCs population play much more important role in humans, and we will pay attention to this issue in future studies.

In this study, we established and compared various gene correction strategies in reporter cells that involved base editors and prime editors. Based on the previously reported information that base editor showed the highest editing efficiency in point mutation,^[^ ^20^, ^24^ ^]^ we first used several ABEs (ABEmax, ABE8e, and CP1041‐ABEmax) to covert the mutant “A” to normal “G” of *Lhcgr* ^W495X^. Among these ABEs, we identified ABE8e displayed the highest base conversion efficiency (37.93% on average) in W495X‐293, however, 91% of the editing outcomes were affected by a bystander (A5). This may owe to that the desired 'A’ (A4) was at the edge of the base editing window of sg1. To overcome this limitation, we tested ABE8e‐NG with sg2 and found that this combination significantly reduced the proportion of bystander effects (reduced to 62.63%), but the gene editing efficiency had also been greatly sacrificed due to the introduction of Cas9‐NG (reduced to 5.45%). Due to the poor efficiency of these established ABE‐based strategies, we tested whether PEs (PE2, PEmax) could effectively correct the point mutation of *Lhcgr* ^W495X^. Finally, PEmax combining with epeg14‐13Tm exhibited high precision rate of gene correction with low level of indels in W495X‐293 reporter cells. In the following experiments, this combination induced 23.42 ± 2.26% “A” to “G” conversion in W495X‐SLCs at 14 days after transduction in vitro. Off‐target analysis didn't detect any undesired edits at predicted potential sites. Similarly, several previous reports showed that PE system has high accuracy of correction.^[^ ^24^, ^58^ ^]^ A recent study also showed the specificity of PE3 system and no guide‐independent off‐target mutations in the DNA or RNA were detected in PE3‐edited cells.^[^ ^59^ ^]^ These results collectively indicated that prime editor is a safe, effective tool and has great potential in gene therapy. We also recognize that off‐targets evaluation of gene editing is critical and will pay attention to this issue in future studies.

In summary, these results suggest that PE‐based therapeutic editing in SLCs ex vivo is a promising strategy for HPH. It is expected that the proof‐of‐concept data will set the stage for further studies and open a new avenue for treating not only HPH but other genetic diseases that affect the reproductive function.

## 4 Experimental Section

### Animals

The *Lhcgr* ^W495X^ mouse line was generated on a C57BL/6 background through CRISPR/Cas9 technology by GemPharmatech (Nanjing, Jiangsu, China). Male mice were identified by PCR and Sanger sequence performed on DNA isolated from the tail (The primers were listed in Table S2, Supporting Information). 8‐week‐old male homozygous *Lhcgr* ^W495X^ mice were randomly assigned to experimental groups. All mice were maintained under controlled temperature (24 ± 1 °C) and relative humidity (50–60%) with a standard 12‐h light‐and‐dark cycle for the duration of the study. All animal experiments were approved by the independent ethics commission (IEC) for Clinical Research and Animal Trials of the First Affiliated Hospital of Sun Yat‐sen University (NO.2021‐297).

### Isolation and Culture of SLCs

Isolation and culture of SLCs were conducted as previously reported by the group.^[^ ^17^ ^]^ In brief, the testes of 7‐day‐old male mice were mechanically cut and enzymatically disassociated with 1 mg mL^−1^ Collagenase IV (Gibco, Grand Island, NY, USA) and 200 µg mL^−1^ DNase I (Gibco) in Dulbecco's modified Eagle medium/nutrient mixture F‐12 (DMEM/F12; 1:1, Gibco) at 37 °C for 15 min with slow shaking (100 cycles min^−1^). The samples were double filtered through a 70 µm strainer and centrifuged at 256 g for 4 min at 4 °C. The cell pellets were rinsed two times with phosphate‐buffered saline (PBS) and then incubated with an anti‐CD51 antibody conjugated with PE (eBiosciences, San Diego, CA, USA) and an isotype antibody in the dark for 15 min. The CD51^+^ SLCs were enriched by flow cytometry (Influx Cell Sorter, BD Biosciences, San Diego, CA, USA) followed by culture in expansion medium consisting of DMEM/F12 (Gibco) supplemented with 2% fetal bovine serum (FBS, SERANA, Pessin, Germany), 1% nonessential amino acids (NEAA, Gibco), 1% insulin‐transferrin‐sodium selenite (ITS, Gibco), 1% N2 (Gibco), 2% B27 (Gibco), 20 ng mL^−1^ basic fibroblast growth factor (PeproTech, NJ, USA), epidermal growth factor (PeproTech), platelet‐derived growth factor‐BB (PeproTech), oncostatin M (PeproTech), 1 ng mL^−1^ leukemia inhibitory factor (Millipore, Bedford, MA, USA), 1 nM dexamethasone (Sigma, St.Louis, MO, USA), and 0.1 mM β‐mercaptoethanol (Gibco). SLCs were seeded into 6‐well plates (NEST, Wuxi, Jiangsu, China) and cultured at 37 °C in a humidified 5% CO_2_ water‐jacketed incubator (Thermo Fisher, Waltham, MA, USA), and the medium was changed every 2 days.

### SLCs Transplantation

For intratesticular injection of PBS or SLCs, a previously reported method was modified.^[^ ^42^ ^]^ Briefly, the mice were anaesthetized with 250 mg kg^−1^ Avertin (Sigma) by intraperitoneal injection. A single incision was made on the ventral skin and body wall ≈1.5 cm anterior to the genitals. The testes were pulled out by holding the fat pad. Care was taken not to injure blood vessels. Each testis was immobilized by fine forceps, SLCs or PBS were injected into the testes using a 33‐gauge needle syringe (Hamilton, Bonaduz, Switzerland). Injection was performed into interstitial spaces (8 µL testis^−1^) and the incision was sutured. For WT‐SLCs transplantation, 8 × 10^4^ cells in 8 µL PBS were injected into each testis of *Lhcgr* ^W495X^ mice. For PE‐W495X‐SLCs or Con‐W495X‐SLCs transplantation, 4 × 10^5^ cells in 8 µL PBS were injected into each testis. The WT mice were subjected to sham surgery as well.

### Construction of Plasmid Vectors

pCMV‐ABEmax‐P2A‐EGFP (Addgene #112 101), ABE8e (Addgene #138 489), pCMV‐PE2‐P2A‐GFP (Addgene #132 776) and pCMV‐PEmax (Addgene #174 820) came from Addgene. To construct pCAG‐ABEmax‐NG‐puro and pCAG‐ABE8e‐NG‐puro, pCMV‐ABEmax‐P2A‐EGFP was digested with MluI‐HF (NEB, Ipswich, MA, USA) and PmeI (NEB). Fragments of CAG promoter, ABEmax‐encoding or ABE8e‐encoding sequences, NG‐Cas9‐encoding sequence (Addgene #124 163) and puro‐encoding sequence were amplified by PCR using SuperFi II DNA Polymerase (Thermo Fisher) and assembled with the linearized plasmid using a ClonExpress Ultra One Step Cloning Kit (Vazyme, Nangjing, Jiangsu, China). To construct pCMV‐CP1041‐ABEmax‐P2A‐EGFP, fragments were gained by PCR from ABEmax, then ligated to linear pCMV‐ABEmax‐P2A‐EGFP digested with NotI‐HF (NEB) and EcoRI‐HF (NEB). The target sequence and puro cassette driven by CAG promoter were inserted into lentiCRISPR v2 backbone (Addgene #52 961) to construct the lentiviral transfer plasmid for generating the reporter HEK293T cell line. To prepare sgRNA plasmids, oligos containing the target sequences were annealed and cloned into pU6 vector. To prepare pegRNA or epegRNA plasmids, oligos containing the target sequence and 3′ extensions were used to amplify pU6 vector and fragments were cloned into pU6‐pegRNA‐GG‐acceptor (Addgene #132 777) or pU6‐pegRNA‐Tm or pU6‐pegRNA‐Te. pLenti‐U6‐epegRNA‐CBh‐PEmax‐N and pLenti‐CBh‐PEmax‐C‐P2A‐EGFP were constructed by inserting corresponding fragments into lentiCRISPR v2 backbone. The pegRNAs, epegRNAs and nicking sgRNAs used in this study were listed in Table S3 (Supporting Information). Sequences of pU6, pU6‐pegRNA‐Tm and pU6‐pegRNA‐Te were provided in Supporting Information.

### In Vitro Transcription of sgRNAs

Templates for in vitro transcription were got through PCR with Phusion Plus DNA polymerase (Thermo Fisher) using primers containing a T7 promoter and target sequences. PCR product was purified with the FastPure Gel DNA Extraction Mini Kit (Vazyme) and transcribed using MEGAshortscript^TM^ Kit (Thermo Fisher). Then, DNA templates were degraded with TURBO^TM^ DNaseI (Thermo Fisher) and transcribed RNAs were purified with MEGAclear^TM^ Kit (Thermo Fisher) according to the manufacturer's protocol. The oligos were listed in Table S2 (Supporting Information).

### Production of Lentivirus

To package lentivirus for construction of HEK293T reporter cell line. HEK293T cells were seeded on a T150 flask (Corning, Corning, NY, USA). At 90% confluency, 18 µg of lentiviral transfer plasmid, 15 µg of psPAX and 10 µg of pMD2.G were co‐transfected using Lipofectamine 3000 (Thermo Fisher). 6 h after transfection, media was replaced. 48 and 72 h after transfection, the supernatant was collected, centrifuged at 3,000 g for 15 min and filtered through a 0.45 µm PVDF membrane (Millipore, Billerica, MA, USA). Then, lentivirus was centrifuged at 50 000 g for 90 min, suspended with 1 mL PBS and stored at −80 °C. To package lentivirus of PEmaxC or PEmaxN, HEK293T cells were seeded on ten T225 flasks (NEST). At 90% confluency, 36 µg of lentiviral transfer plasmid, 27 µg of psPAX and 18 µg of pMD2.G were co‐transfected into each T225 flask using Lipofectamine 3000. Lentivirus particles were collected, purified and concentrated as mentioned above. Finally, lentivirus was suspended with 6 mL culture medium of SLCs, divided into 200 µL per tube and stored at −80 °C.

### Cell Culture, Transfection, and Genomic DNA Extraction of HEK293T Cell

HEK293T was cultured in DMEM (Corning) with 10% FBS, 1% Penicillin‐Streptomycin (Gibco) and 1% GlutaMAX (Gibco). 8 × 10^4^ cells per well were seeded on 48‐well plates (NEST) and transfected at 70% confluence with 1 µL Lipofectamine 3000 plus 1 µL P3000 according to the manufacturer's protocol. For base editing, 500 ng ABEs plasmid and 500 ng in vitro transcribed sgRNA were used per well. For prime editing, 500 ng PEs plasmid, 125 ng pegRNA, or epegRNA plasmid and with or without 42 ng nicking sgRNA plasmid (PE3 or PE2) were used per well. 72 h after transfection, genomic DNA was isolated. In brief, medium was discarded, and cells were lysed with lysis buffer (10 mM Tris‐HCl, pH 7.5 (Macklin, Shanghai, China), 0.05% SDS (Macklin), 800 units/L proteinase K (NEB) at 37 °C for 1 h, then protease K was inactivated at 80 °C for 20 min.

### Construction of HEK‐293 Cell Line with Integrated *Lhcgr* ^W495X^ Target Sequence

HEK293T cells were seeded on a T75 flask and transduced with lentivirus containing *Lhcgr* ^W495X^ target sequence with 8 µg mL^−1^ polybrene. 48 h after transduction, infected cells were cultivated in the culture with 2 µg mL^−1^ puromycin for the following 7 days.

### Lentivirus Infection of SLCs

WT‐SLCs at passage 1 were transduced with mCherry lentivirus (PackGene Biotech, Guangzhou, China) at 30% confluence in 6‐well plates. Three days after transfection, the cells were collected and centrifuged at 250 g at 4 °C for 4 min, and the pellet was washed twice with PBS. The mCherry^+^ cells were enriched by flow cytometry using an Influx Cell Sorter (BD Bioscience).

W495X‐SLCs at passage 1 were transduced with 200 µL PEmaxN and 200 µL PEmaxC lentivirus or only transduced with 200 µL PEmaxC lentivirus (as control) at 60% confluence in 6‐well plates with the presence of 8 µg mL^−1^ polybrene. 12 h after transduction, media was refreshed. SLCs were digested with Accutase (Gibco) and transplanted into testes of *Lhcgr* ^W495X^ mice.

### High‐Throughput Sequencing and Off‐Target Analysis

To evaluate the editing efficiency of various editing strategies, target sequences were amplified via two rounds of PCR. The first PCR using primers containing inline‐barcodes (Table S4, Supporting Information) and Illumina adapter sequences. Then, 1 µL the first PCR product was used as the template for the second PCR using primers containing unique i7 and i5 Illumina barcode combinations. Finally, the products were pooled, gel‐purified with FastPure Gel DNA Extraction Mini Kit (Vazyme DC301) and analyzed with the NovaSeq 6000. 4 top‐ranking predicted off‐target sites were analyzed and all predicted off‐target sites for 3 nt mismatch from CRISPOR through deep sequencing.^[^ ^40^ ^]^ All the HTS data were analyzed using CRISPResso2.^[^ ^60^ ^]^ All primers used in HTS were listed in Table S2 (Supporting Information).

### In Vitro Differentiation of SLCs

For LCs differentiation, a previously method reported by the group was applied.^[^ ^17^ ^]^ In brief, the SLCs were induced in DIM containing M199 Medium (Gibco), 4% FBS, 1% ITS (Gibco), 1% GlutaMAX (Gibco), 10 ng mL^−1^ platelet‐derived growth factor‐AA (PDGF‐AA, PeproTech), 0.25 µM smoothened agonist (Sigma), 10 ng mL^−1^ LH (Sigma), 50 µg mL^−1^ insulin‐like growth factor 1 (IGF‐1, PeproTech). The cell supernatants were collected for evaluation of testosterone 2 weeks after induction. The differentiation was confirmed by immunostaining and quantitative RT‐PCR for LCs lineage‐specific markers (Primers and antibodies were listed in Table S2 and S5, Supporting Information, respectively).

### RNA Extraction, cDNA Synthesis, and Quantitative RT‐PCR

Total RNA from the testes or cells was extracted using a RNeasy mini kit (Qiagen, Dusseldorf, Germany) according to the manufacturer's protocol. Reverse transcription was performed using a High‐Capacity RNA‐to‐cDNA^TM^ Kit (Thermo Fisher). Quantitative RT‐PCR was performed using SYBR PCR Master Mix (Roche, Basel, Switzerland) and a Light Cycler 480 Detection System (Roche). To check primers, a melting curve was generated to confirm a single peak and rule out the possibility of non‐specific product or primer‐dimer formation. β‐actin was amplified as internal control, and the target gene expression was presented as ratio to WT mice or WT‐SLCs. The primers used for quantitative RT‐PCR were listed at Table S2 (Supporting Information).

### Immunofluorescence Staining

For immunofluorescence staining of testes, the tissues were fixed in 4% paraformaldehyde (PFA, Phygene, Fuzhou, Fujian, China) at 4 °C overnight, dehydrated with 30% sucrose (Sangon Biotech, Shanghai, China), and sectioned at a thickness of 10 µm. The sections were permeabilized for 15 min using 0.2% Triton X‐100 (Sigma), blocked with 2% bovine serum albumin (BSA, Sigma) and 10% goat serum (Boster, Wuhan, Hubei, China) in PBS for 45 min at room temperature and then incubated overnight with the primary antibodies. The sections were then washed with PBS for 3 times, incubated with the appropriate secondary antibodies for 45 min at room temperature, and co‐stained with DAPI (Gibco) for 5 min. Images was captured with a LSM800 confocal microscope (Zeiss, Oberkohen, Germany) or a Leica DMi8 microscope (Leica, Solms, Germany). The primary and secondary antibodies were listed in Table S5 (Supporting Information).

For immunofluorescence analysis, cells were seeded in 24‐well plate. They were fixed in 4% PFA for 15 min. The fixed cells were treated with PBS containing 0.2% Triton X‐100 for 15 min at room temperature. Then, non‐specific binding of antibodies was blocked with 2% BSA and 10% goat serum in PBS for 45 min at room temperature and incubated overnight with relevant primary antibodies at 4 °C. Next, cells were washed with PBS 3 times and then incubated with the appropriate secondary antibodies for 45 min at room temperature. The nuclei were counterstained with DAPI for 5 min. Images was captured with a LSM800 confocal microscope (Zeiss, Oberkohen, Germany) or a Leica DMi8 microscope (Leica, Solms, Germany). The primary and secondary antibodies were listed in Table S5 (Supporting Information).

### Hematoxylin and Eosin (H&E) Staining

The testes and epididymides were collected, fixed overnight in Bouin's solution (Sigma), dehydrated in 75% ethanol, embedded in paraffin, and sectioned at 4 µm. The sections were deparaffinized with xylene, hydrated with graded ethanol, and stained with hematoxylin and eosin for histological analysis using a Leica DMi8 microscope.

### Sex Hormone Assays

Sex hormone concentrations were assayed as previously reported by the group.^[^ ^42^ ^]^ Serum and testes samples were collected at the indicated timepoints and stored at −80 °C until analysis. Testosterone levels were measured using a chemiluminescent immunoassay (CLIA) by commercial company (KingMed Diagnostics Group Co., Ltd., Guangzhou, Guangdong, China). The concentration of serum LH and FSH were detected using ELISA kits (Cloud‐Clone, Wuhan, Hubei, China) according to the manufacturer's protocol.

### Anogenital Distance Measurement

AGD was measured at indicated timepoints as previously described.^[^ ^61^ ^]^ Briefly, mice were positioned on a horizontal surface and gently lift their tail to have a clear access to the genitalia. Then, the distance was measured from the proximal end of the anus to the base of the genitals using an adapted ruler. Record each measurement for subsequent statistical analysis.

### Computer‐Aided Semen Analysis

Semen samples were analyzed as previously reported.^[^ ^42^ ^]^ In brief, two cauda epididymides were harvested from each mouse, incised with micro scissors, and incubated in 0.5 mL DMEM/F12 containing 0.5% BSA for 15 min at 37 °C to allow for sperm release. The tissue was removed, and sperm samples were analyzed using a Hamilton Thorne's Ceros II system. At least six fields were assessed for each sample, and the sperm concentration and percentages of motile spermatozoa were determined by average value of two cauda epididymides.

### In Vitro Fertilization (IVF) and Mouse Embryo Transfer

IVF was conducted as previously described.^[^ ^42^ ^]^ Sperm were collected from the cauda epididymis of *Lhcgr* ^W495X^ mice at 6 weeks after PE‐W495X‐SLCs injection, and then incubated in a humidified 5% CO2 water‐jacketed incubator (Thermo Fisher) at 37 °C for 1.5 h in a 100 µL drop of TYH medium for capacitation. Cumulus‐oocyte complexes were collected from the oviducts of WT female C57BL/6 mice at 16 h after ovulatory hCG (Sigma) injection and placed in a 60 µL drop of HTF medium that was covered by mineral oil (Sigma). IVF was performed by adding capacitated sperm (≈12000 sperm/60 µL) into the medium containing the oocytes. After 4 h, the oocytes were collected and transferred to KSOM medium and cultured in a humidified 5% CO2 water‐jacketed incubator (Thermo Fisher) at 37 °C. The numbers of 2‐cell embryos were determined at 21 h after insemination. The 2‐cell embryo formation rate was calculated by dividing the number of 2‐cell embryos by the number of oocytes examined. For embryo transfer, 8‐ to 12‐week‐old females were mated with vasectomized males and determined whether they were pseudo‐pregnant by monitoring vaginal plugs. Then, 2‐cell embryos were surgically transferred into the oviducts of pseudo‐pregnant females. The numbers of pups were determined at 20 days after embryos transfer. The pups production rate was calculated by dividing the number of pups by the number of 2‐cell embryos transferred.

### Statistical Analysis

All data were presented as box plots with minimum to maximum values or mean ± SD. Data were analyzed using SPSS Statistics software (IBM SPSS Statistics, Armonk, NY, USA). A two‐tailed *t*‐test or Mann‐Whitney U tests was used for two‐group comparisons. One‐way and two‐way analysis of variance (ANOVA) were used for comparisons of three groups. Differences were considered significant when P < 0.05 (* *p* < 0.05, ** *p* < 0.01 and *** *p* < 0.001), ns = not significant.

## Conflict of Interest

The authors declare no conflict of interest.

## Author Contributions

K.X., F.W., Z.T., S.Z. contributed equally to this work. K.X. carried out the experiments, assisted with the experimental design and wrote the manuscript. F.W., Z.T., and S.Z. carried out the experiments and data analysis. X.L. assisted with the design of *Lhcgr^W495X^* mouse model. C.Y. and W.O. performed animal experiments. A.H. and H.P. assisted with animal experiments. H.C. and P.L. helped with immunofluorescence staining. X.T., T.W. and Q.K. assisted with the experimental design and revised the manuscript. A.P.X. and C.D. conceived the project, supervised all experiments, and wrote and revised the manuscript. All authors read and approved the final version of the paper.

## Supporting information

Supporting Information

Supplemental Table 1

Supplemental Table 2

Supplemental Table 3

Supplemental Table 4

Supplemental Table 5

## Data Availability

The data that support the findings of this study are openly available in NCBI Sequence Read Archive at https://www.ncbi.nlm.nih.gov/bioproject/PRJNA853891, reference number 853891.
