# Supplementary material for: Precise Correction of Lhcgr Mutation in Stem Leydig Cells by Prime Editing Rescues Hereditary Primary Hypogonadism in Mice
Source: Adv Sci (Weinh). 2023 Sep 11;10(29):2300993. doi: 10.1002/advs.202300993 (PMC10582410; doi:10.1002/advs.202300993)
Supplement: Supplementary file 1 — Supporting Information [file ADVS-10-2300993-s001.pdf]

ADVANCED SCIENCE
Open Access

Supporting Information

for *Adv. Sci.*, DOI 10.1002/advs.202300993

Precise Correction of *Lhcgr* Mutation in Stem Leydig Cells by Prime Editing Rescues Hereditary Primary Hypogonadism in Mice

Kai Xia, Fulin Wang, Zhipeng Tan, Suyuan Zhang, Xingqiang Lai, Wangsheng Ou, Cuifeng Yang, Hong Chen, Hao Peng, Peng Luo, Anqi Hu, Xiang'an Tu, Tao Wang, Qiong Ke, Chunhua Deng and Andy Peng Xiang**

Supporting Information

Precise Correction of Lhcgr Mutation in Stem Leydig Cells by Prime Editing Rescues Hereditary Primary Hypogonadism in Mice

Kai Xia, Fulin Wang, Zhipeng Tan, Suyuan Zhang, Xingqiang Lai, Wangsheng Ou, Cuifeng Yang, Hong Chen, Hao Peng, Peng Luo, Anqi Hu, Xiang'an Tu, Tao Wang, Qiong Ke, Chunhua Deng, Andy Peng Xiang**

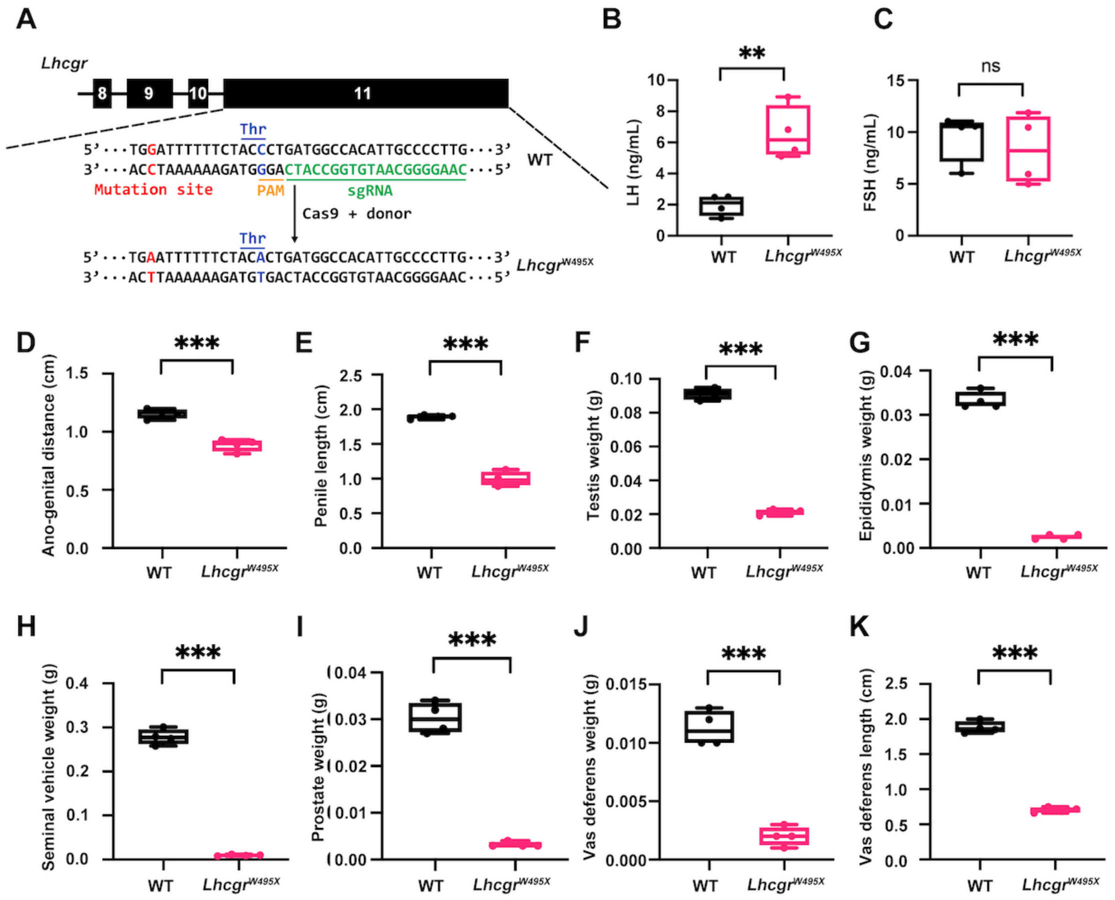

Figure S1. Generation strategy and phenotypes of *Lhcgr*^{W495X} knock-in point mutation mice. A) Strategy for the generation of the *Lhcgr*^{W495X} knock-in point mutation mice. The nonsense mutation site is indicated in red. The synonymous mutation introduced for destruction of PAM is indicated in blue. PAM and sgRNA are represented by green and orange lines respectively. B,C) LH (B) and FSH (C) level of 8-week-old WT and *Lhcgr*^{W495X} mice (n=4). D-K) The length of ano-genital distance (D), penile (E) and the weight of testis (F), epididymis (G), seminal vesicle (H), prostate (I), vas deference (J) and the length of vas deference (K) of 8-week-old WT and *Lhcgr*^{W495X} mice (n=4). Data are represented by box plots, and whiskers are minimum to maximum values. Significance was determined by two-tailed unpaired *t*-test. ** P < 0.01, *** P < 0.001, ns = not significant.

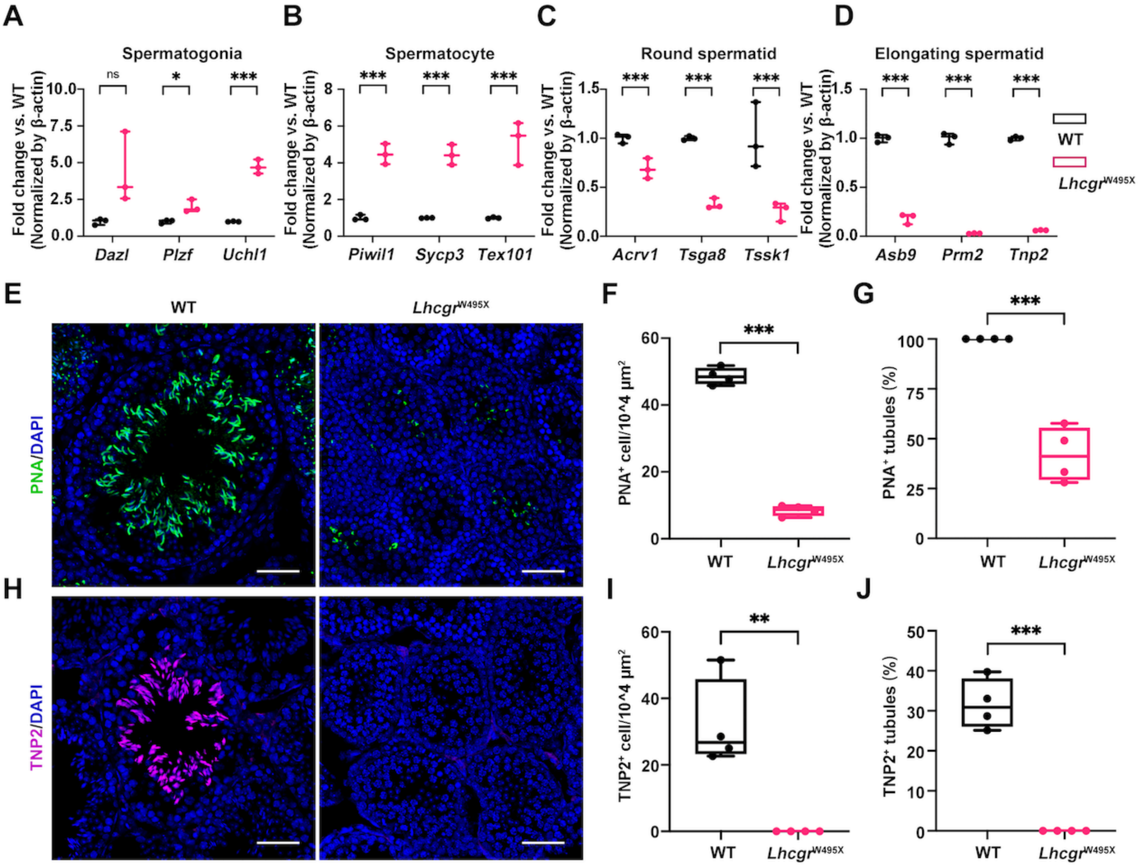

Figure S2. Characterization of spermatogenesis of *Lhcgr*^{W495X} mice.

A-D) Quantitative RT-PCR analysis of spermatogonia (*Dazl*, *Plzf*, and *Uchl1*, A), spermatocyte (*Piwil1*, *Sycp3*, and *Tex101*, B), round spermatid (*Acrv1*, *Tsga8*, and *Tssk1*, C), and elongating spermatid (*Asb9*, *Tnp2*, and *Prm2*, D) in WT and *Lhcgr*^{W495X} mice (n=4), β-actin was used as internal control. E-G) Immunofluorescence staining of acrosome marker PNA (E) in testes sections, and quantitative analysis the number of PNA$^+$ cells in 10^4 μm^2 area (F) and the percentage of PNA$^+$ tubules (G) from indicated groups (n=4). Three sections per slide and three slides per testis were randomly selected and evaluated. Scale bars: 50 μm. H-J) Immunofluorescence staining of elongating spermatid marker TNP2 (H) in testes sections, and quantitative analysis the number of TNP$^+$ cells in 10^4 μm^2 area (I) and the percentage of TNP2$^+$ tubules (J) from indicated groups (n=4). Three sections per slide and three slides per testis were randomly selected and evaluated. Scale bars: 50 μm. Data are represented by box plots, and whiskers are minimum to maximum values. Significance was determined by two-tailed unpaired *t*-test. * P < 0.05, ** P < 0.01, *** P < 0.001, ns = not significant.

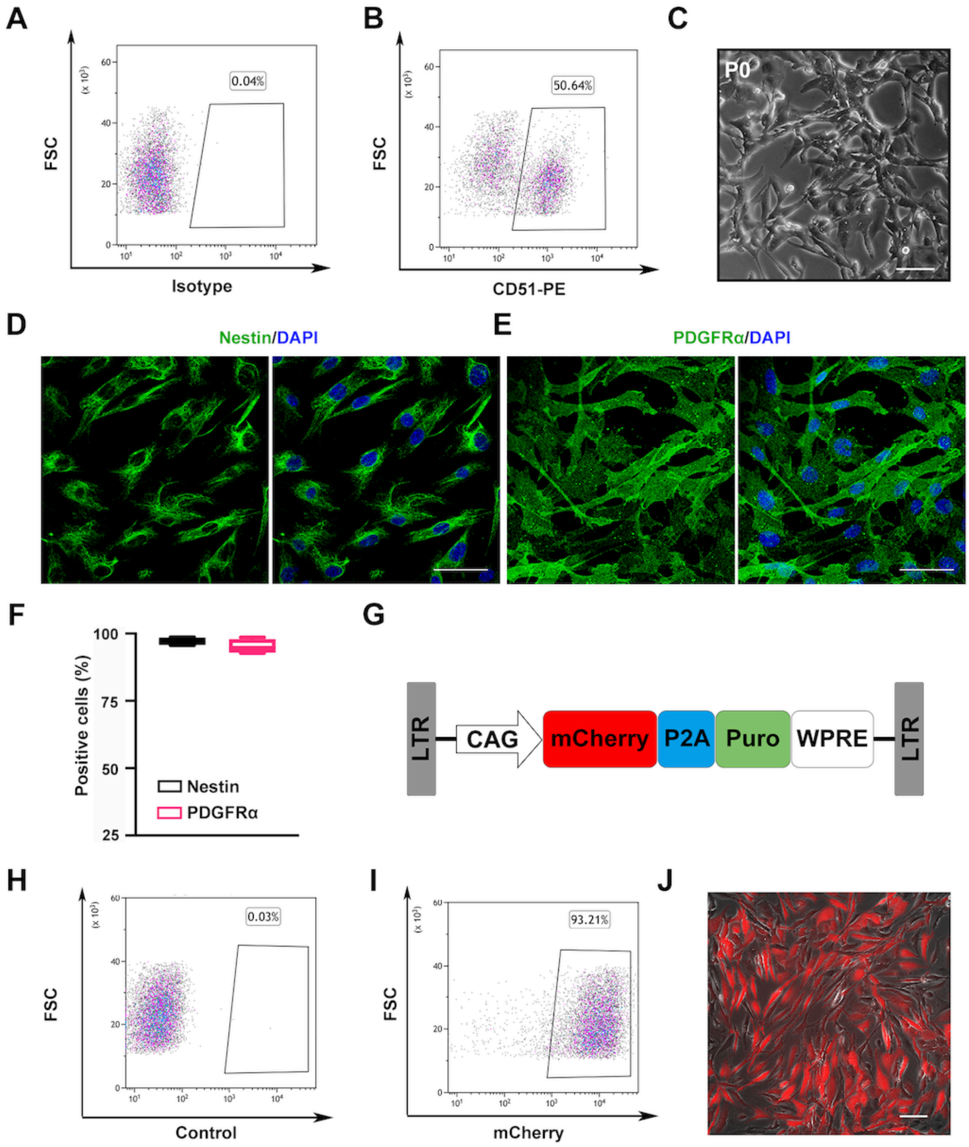

Figure S3. Isolation and transfection of WT-SLCs.

A,B) $CD51^{+}$ SLCs were isolated by FACS from testes of 7-day-old WT mice (n=4). A: isotype controls. B: stained samples. FSC: forward scatter. C) Representative image of the isolated $CD51^{+}$ SLCs at primary passage (P0, n=4). Scale bars: 75 µm. D,E) $CD51^{+}$ SLCs expressed Nestin (D) and PDGFRα (E). Scale bars: 50 µm. F) Quantitative analysis the proportion of $Nestin^{+}$ cells and $PDGFR\alpha^{+}$ cells in total sorted $CD51^{+}$ cells (n=4). G) Schematic view of the mCherry lentivirus. H,I) $mCherry^{+}$ cells were sorted by FACS. J) Representative images of the $mCherry^{+}$ SLCs. Scale bar: 100 µm. Data are represented by box plots, and whiskers are minimum to maximum values.

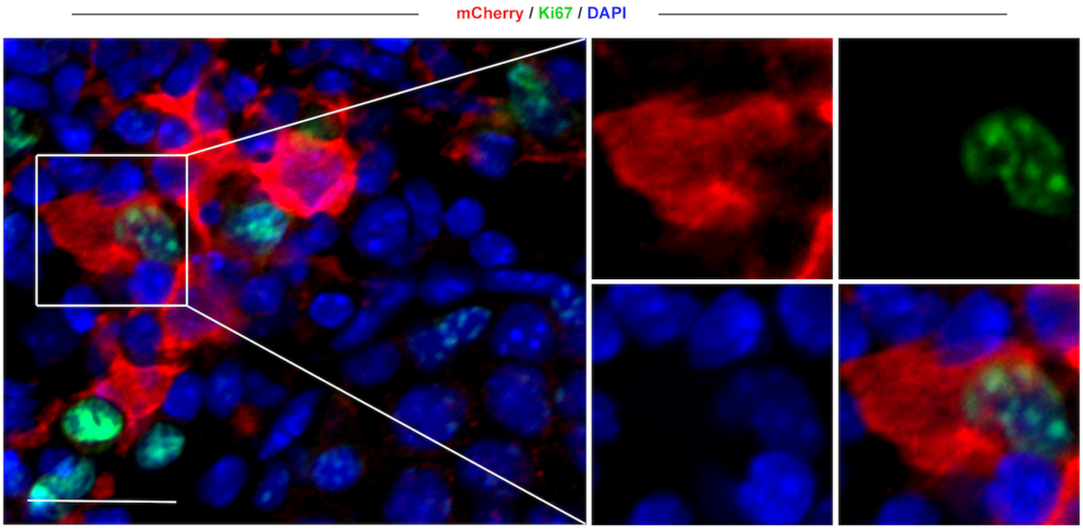

Figure S4. The proliferation of transplanted WT-SLCs.

Proliferation of the transplanted WT-SLCs, as demonstrated by staining for Ki67. The nuclei were counter-stained with DAPI. The images represent the results obtained from recipient mice (n=3). mCherry indicates transplanted SLCs. Scale bar: 20 μm.

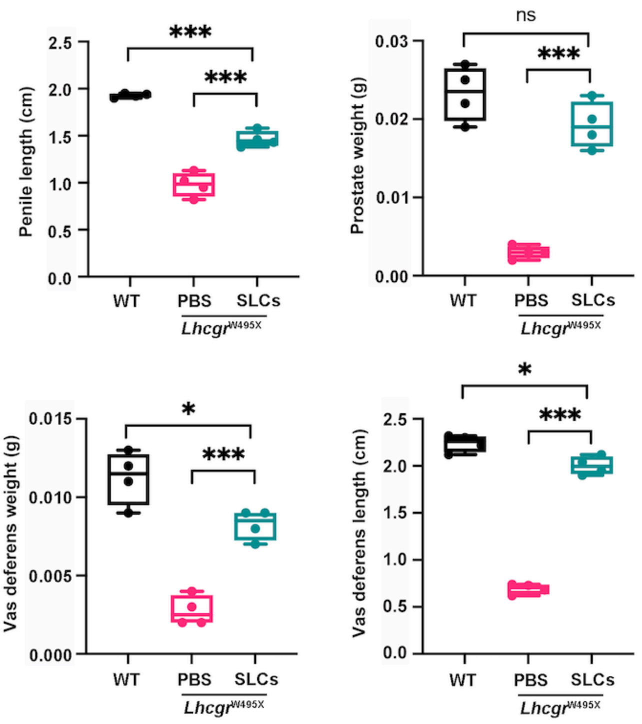

Figure S5. Quantitative analysis of the genital phenotype in *Lhcgr*^{W495X} mice at 4 weeks after WT-SLCs transplantation.

The length of penile, the weight of prostate, the weight of vas deference and the length of vas deference of WT and *Lhcgr*^{W495X} mice injected with PBS or SLCs (8×10^4 cells/testes) at 4 weeks after treatment (n=4). Data are represented by box plots, and whiskers are minimum to maximum values. Significance was determined by one-way ANOVA. * P < 0.05, *** P < 0.001, ns = not significant.

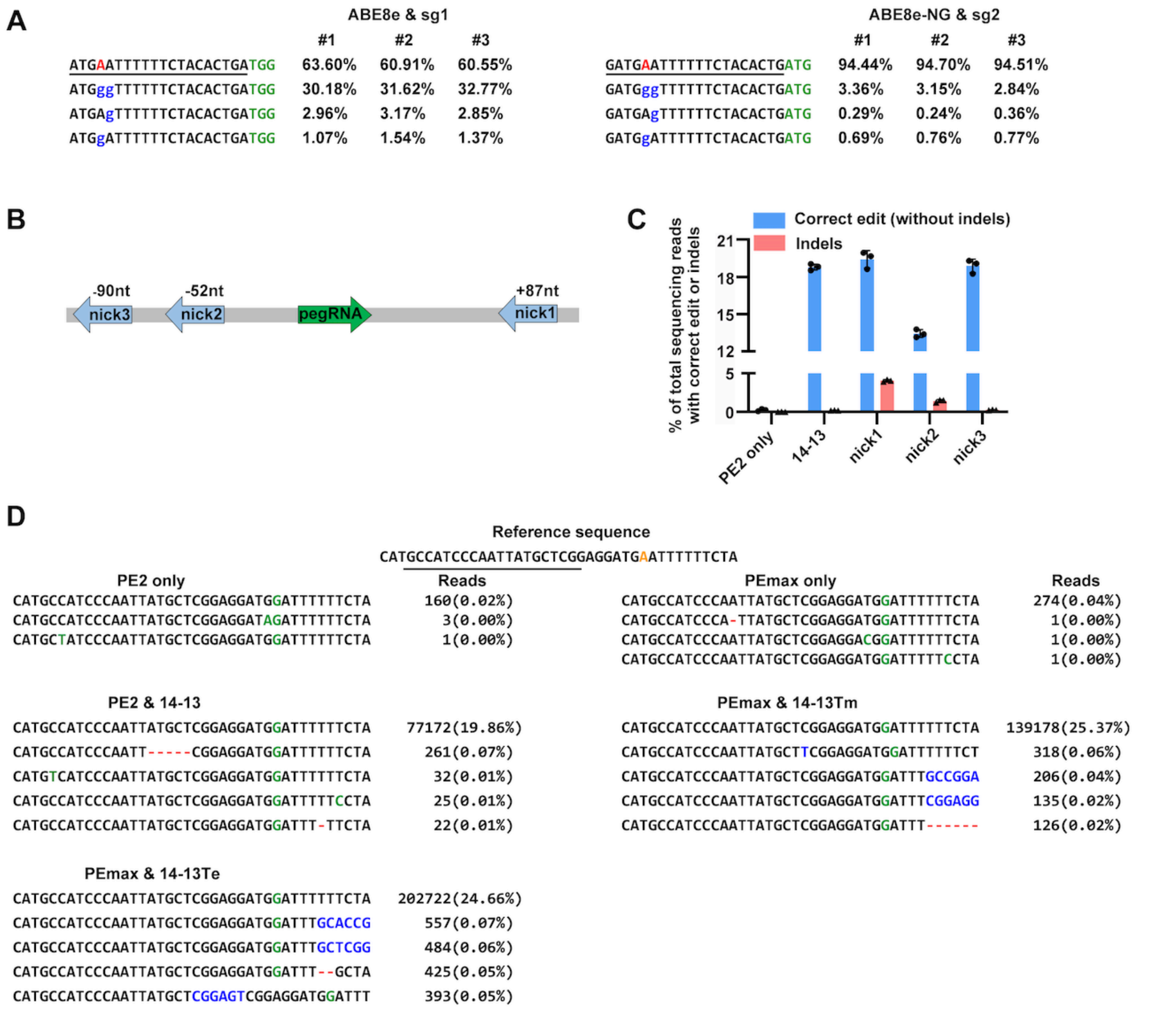

Figure S6. Supplementary information about base editing and prime editing.

A) DNA sequences of W495X-293 edited by ABE8e & sg1 or ABE8e-NG & sg2 are presented. The WT sequence is underlined, target 'A' (red), edited sequences (blue) and PAM (green) are highlighted. B) Schematic view of pegRNA and nicking sgRNA target sites. The nicking position is marked next to each nicking sgRNA. C) The editing efficiencies of PE2 and PE3 containing three different nicking sgRNAs. Indels are plotted as red column. Data are presented by mean ± SD of independent transfections (n=3). D) Representative prime-edited results around PE-spacer-sgRNA. The targeted position in reference sequence is shown in orange and the PE-spacer-sgRNA is underlined. DNA sequences and the read count of sequences are shown. Insert sequences are shown in blue, deletion sequences in red, and base substitution in green.

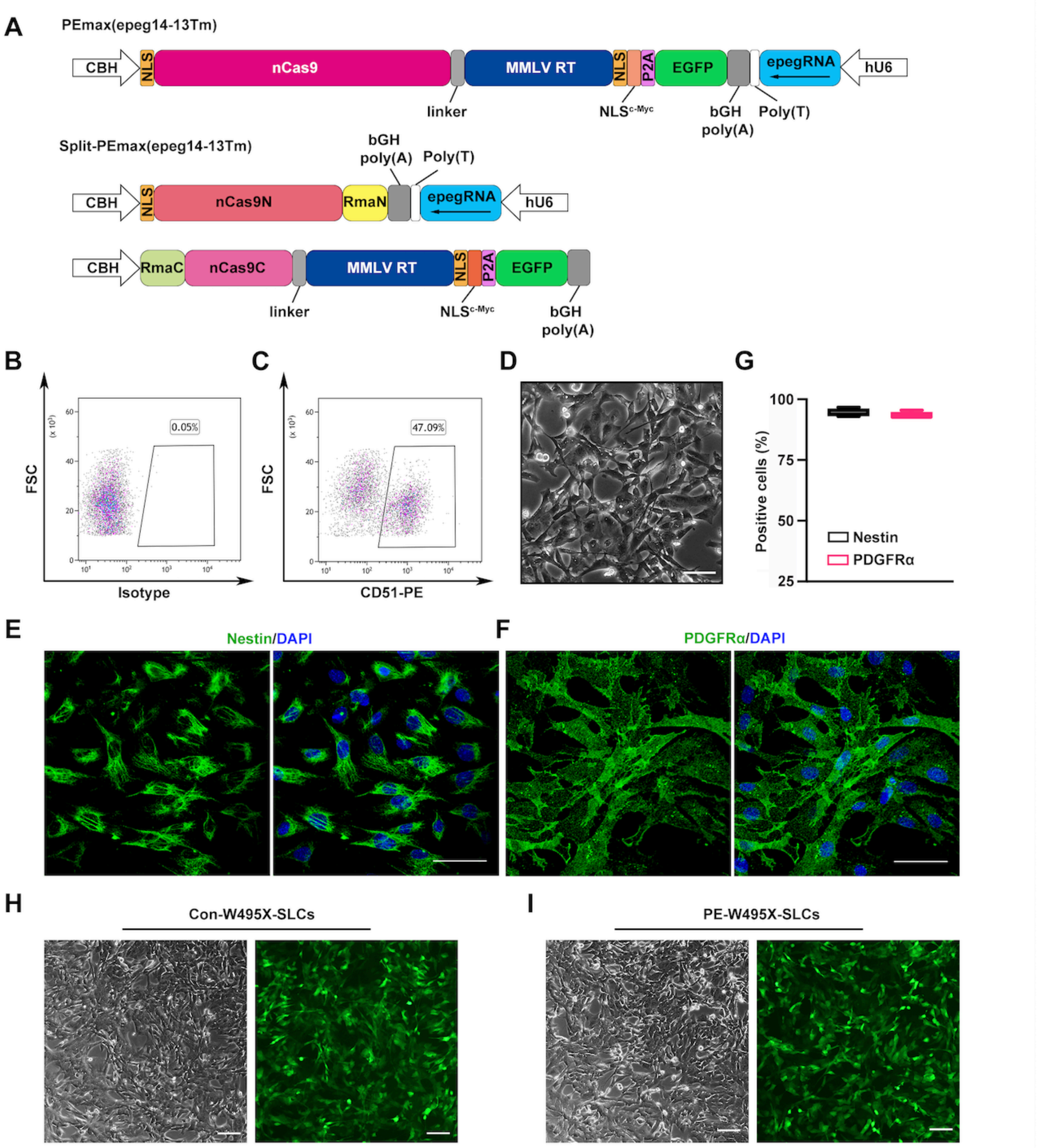

Figure S7. Transduction of W495X-SLCs with PEmax combining with epeg14-13Tm. A) Schematic view of vectors encoding Full-length PEmax combining with epeg14-13Tm and Split-PEmax combining with epeg14-13Tm. B,C) $CD51^{+}$ SLCs were isolated by FACS from testes of 7-day-old *Lhcgr*^{W495X} mice (n=4). B: Isotype, C: stained samples. FSC: forward scatter. D) Representative image of the isolated $CD51^{+}$ SLCs at P0 (n=4). Scale bar: 75 µm. E,F) The sorted $CD51^{+}$ SLCs expressed Nestin (E) and PDGFRα (F). Scale bars: 50 µm. G) Quantitative analysis the proportion of $Nestin^{+}$ and $PDGFR\alpha^{+}$ cells in sorted $CD51^{+}$ SLCs (n=4). H,I) Representative micrographs showing EGFP protein expression in W495X-SLCs 48h after PEmaxC (Con-W495X-SLC, H) or Split-PEmax combining with epeg14-13Tm (PE-W495X-SLC, I) lentivirus transduction. Scale bars: 100 µm.

Reference sequence

CATGCCATCCCAATTATGCTCGGAGGATGAATTTTTTCTA

con	Reads
CATGCCATCCCAATTATGCTCGGAGGATGGATTTTTTCTA	471(0.04%)
CATGCCATCCCAATTATGCTCGGAGGATGGATTTTTTTTCT	1(0.00%)
CATGCCATCCCAATTATGCTTGGAGGATGGATTTTTTCTA	1(0.00%)
CATGTCATCCCAATTATGCTCGGAGGATGGATTTTTTCTA	1(0.00%)

7d	Reads
CATGCCATCCCAATTATGCTCGGAGGATGGATTTTTTCTA	108788(11.84%)
CATGCCATCCCAATTATGCTCTCGGAGGATGGATTTTTTC	767(0.08%)
CATGCCATCCCAATTATGCTCGGAGGATGTCGGAGGATGG	694(0.08%)
CATGCCATCCCAATTATGCTCGGAGGATGGATTTGTTCTA	517(0.06%)
CATGCCATCCCAATTATGCTCAGAGGATGGATTTTTTCTA	61(0.01%)

14d	
CATGCCATCCCAATTATGCTCGGAGGATGGATTTTTTCTA	266328(24.63%)
CATGCCATCCCAATTATGCTCGGCGGAGGATGGATTTTTT	1323(0.12%)
CATGCCATCCCAATTATGCTCGGAGAATGGATTTTTTCTA	1059(0.10%)
CATGCCATCCCAATTATGCTCGGAGGATGGATTTGCTCTA	1020(0.09%)
CATGCCATCCCAATTATGCTCGGAGTCGGAGGATGGATTT	828(0.08%)

Figure S8. Representative prime-edited results in W495X-SLC.

The targeted position in reference sequence is shown in orange and the PE-spacer-sgRNA is underlined. DNA sequences and the read count of sequences are shown. Insert sequences are shown in blue, and base substitution in green.

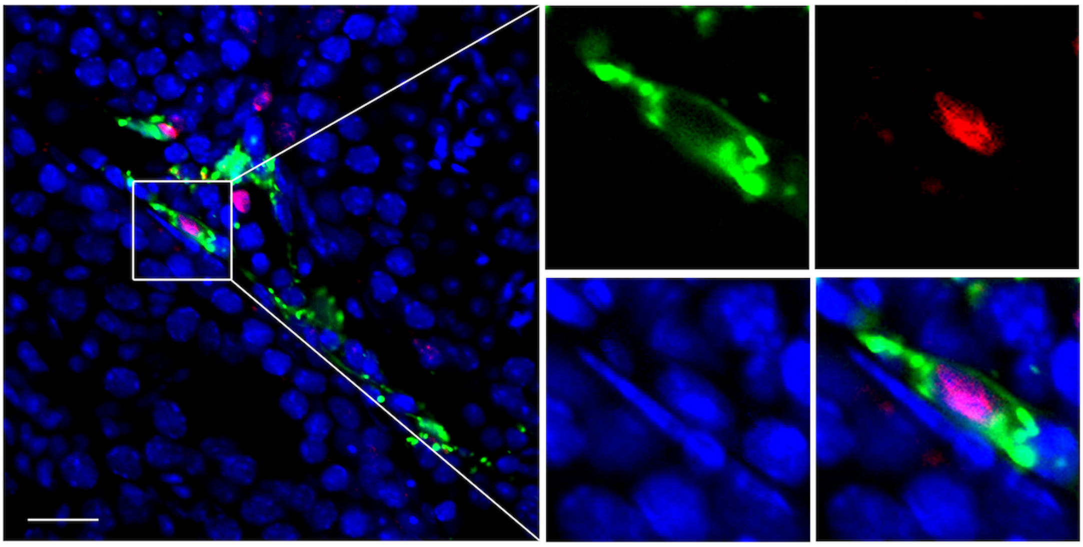

Figure S9. The proliferation of transplanted PE-W495X-SLCs.

Proliferation of the transplanted PE-W495X-SLCs, as demonstrated by staining for Ki67. The nuclei were counter-stained with DAPI. The images represent the results obtained from recipient mice (n=3). EGFP indicates transplanted PE-W495X-SLCs. Scale bar: 20 µm.

Supplementary Sequences. Sequences of pU6, pU6-pegRNA-Tm and pU6-pegRNA-Te.

pU6:

gagggcctatttcccatgattccttcatatttgcatatacgatacaaggctgttagagagataattggaattaatttgactgtaaacacaaag
atattagtacaaaatacgtgacgtagaaagtaataatttcttgggtagtttgcagttttaaaattatgttttaaaatggactatcatatgcttacc
gtaacttgaaagtatttcgatttcttggctttatatatcttgtggaaaggacgaaacaccgggtcttcgagaagacctgttttagagctagaa
atagcaagttaaaataaggctagtccgttatcaacttgaaaaagtggcaccgagtcggtgcttttttaagcttggcgtaatcatggtcatag
ctgtttcctgtgtgaaattgttatccgctcacaattccacacaacatacgagccggaagcataaagtgtaaagcctggggtgcctaatgag
tgagctaactcacattaattgcgttgcgctcactgcccgctttccagtcgggaaacctgtcgtgccagctgcattaatgaatcggccaacg
cgcggggagaggcggtttgcgtattgggcgctcttccgcttcctcgctcactgactcgctgcgctcggtcgttcggctgcggcgagcg
gtatcagctcactcaaaggcggtaatacggttatccacagaatcaggggataacgcaggaaagaacatgtgagcaaaaggccagcaa
aaggccaggaaccgtaaaaaggccgcgttgctggcgtttttccataggctccgcccccctgacgagcatcacaaaaatcgacgctcaa
gtcagaggtggcgaaacccgacaggactataaagataccaggcgtttccccctggaagctccctcgtgcgctctcctgttccgaccct
gccgcttaccggatacctgtccgcctttctcccttcgggaagcgtggcgctttctcatagctcacgctgtaggtatctcagttcggtgtagg
tcgttcgctccaagctgggctgtgtgcacgaaccccccgttcagcccgaccgctgcgccttatccggtaactatcgtcttgagtccaacc
cggtaagacacgacttatcgccactggcagcagccactggtaacaggattagcagagcgaggtatgtaggcggtgctacagagttctt
gaagtggtggcctaactacggctacactagaagaacagtatttggtatctgcgctctgctgaagccagttaccttcggaaaaagagttgg
tagctcttgatccggcaaacaaaccaccgctggtagcggtggtttttttgtttgcaagcagcagattacgcgcagaaaaaaaggatctca
agaagatcctttgatcttttctacggggtctgacgctcagtggaacgaaaactcacgttaagggattttggtcatgagattatcaaaaagg
atcttcacctagatccttttaaattaaaaatgaagttttaaatcaatctaaagtatatatgagtaaacttggtctgacagttaccaatgcttaatc
agtgaggcacctatctcagcgatctgtctatttcgttcatccatagttgcctgactccccgtcgtgtagataactacgatacgggagggctt
accatctggccccagtgctgcaatgataccgcgagacccacgctcaccggctccagatttatcagcaataaaccagccagccggaag
ggccgagcgcagaagtggtcctgcaactttatccgcctccatccagtctattaattgttgccgggaagctagagtaagtagttcgccagtt
aatagtttgcgcaacgttgttgccattgctacaggcatcgtggtgtcacgctcgtcgtttggtatggcttcattcagctccggttcccaacg
atcaaggcgagttacatgatcccccatgttgtgcaaaaaagcggttagctccttcggtcctccgatcgttgtcagaagtaagttggccgc
agtgttatcactcatggttatggcagcactgcataattctcttactgtcatgccatccgtaagatgcttttctgtgactggtgagtactcaacc
aagtcattctgagaatagtgtatgcggcgaccgagttgctcttgcccggcgtcaatacgggataataccgcgccacatagcagaacttta
aaagtgctcatcattggaaaacgttcttcggggcgaaaactctcaaggatcttaccgctgttgagatccagttcgatgtaacccactcgtg
cacccaactgatcttcagcatcttttactttcaccagcgtttctgggtgagcaaaaacaggaaggcaaaatgccgcaaaaaagggaata
agggcgacacggaaatgttgaatactcatactcttcctttttcaatattattgaagcatttatcagggttattgtctcatgagcggatacatatt
tgaatgtatttagaaaaataaacaaataggggttccgcgcacatttccccgaaaagtgccacctgacgtctaagaaaccattattatcatg
acattaacctataaaaataggcgtatcacgaggccctttcgtctcgcgcgtttcggtgatgacggtgaaaacctctgacacatgcagctc
ccggagacggtcacagcttgtctgtaagcggatgccgggagcagacaagcccgtcagggcgcgtcagcgggtgttggcgggtgtc
ggggctggcttaactatgcggcatcagagcagattgtactgagagtgcaccatatgcggtgtgaaataccgcacagatgcgtaaggag
aaaataccgcatcaggcgccattcgccattcaggctgcgcaactgttgggaagggcgatcggtgcgggcctcttcgctattacgccag

ctggcgaaagggggatgtgctgcaaggcgattaagttgggtaacgccagggttttcccagtcacgacgttgtaaaacgacggccagt gaattcgagctcggtacccggggatccgttaattaa

pU6-pegRNA-Tm:

gagggcctatttcccatgattccttcatatttgcatatacgatacaaggctgttagagagataattagaattaatttgactgtaaacacaaag atattagtacaaaatacgtgacgtagaaagtaataatttcttgggtagtttgcagttttaaaattatgttttaaaatggactatcatatgcttacc gtaacttgaaagtatttcgatttcttggctttatatatcttgtggaaaggacgaaacaccgagaccgaggtctcggggtcaggagcccccc cccctgaacccaggataaccctcaaagtcggggggctttttttaagcttgggccgctcgaggtacctctctacatatgacatgtgagcaa aaggccagcaaaaggccaggaaccgtaaaaaggccgcgttgctggcgtttttccataggctccgcccccctgacgagcatcacaaaa atcgacgctcaagtcagaggtggcgaaacccgacaggactataaagataccaggcgtttccccctggaagctccctcgtgcgctctcc tgttccgaccctgccgcttaccggatacctgtccgcctttctcccttcgggaagcgtggcgctttctcatagctcacgctgtaggtatctca gttcggtgtaggtcgttcgctccaagctgggctgtgtgcacgaaccccccgttcagcccgaccgctgcgccttatccggtaactatcgtc ttgagtccaacccggtaagacacgacttatcgccactggcagcagccactggtaacaggattagcagagcgaggtatgtaggcggtg ctacagagttcttgaagtggtggcctaactacggctacactagaagaacagtatttggtatctgcgctctgctgaagccagttaccttcgg aaaaagagttggtagctcttgatccggcaaacaaaccaccgctggtagcggtggtttttttgtttgcaagcagcagattacgcgcagaaa aaaaggatctcaagaagatcctttgatcttttctacggggtctgacgctcagtggaacgaaaactcacgttaagggattttggtcatgaga ttatcaaaaaggatcttcacctagatccttttaaattaaaaatgaagttttaaatcaatctaaagtatatatgagtaaacttggtctgacagtta ccaatgcttaatcagtgaggcacctatctcagcgatctgtctatttcgttcatccatagttgcctgactccccgtcgtgtagataactacgat acgggagggcttaccatctggccccagtgctgcaatgataccgcgagatccacgctcaccggctccagatttatcagcaataaaccag ccagccggaagggccgagcgcagaagtggtcctgcaactttatccgcctccatccagtctattaattgttgccgggaagctagagtaag tagttcgccagttaatagtttgcgcaacgttgttgccattgctacaggcatcgtggtgtcacgctcgtcgtttggtatggcttcattcagctcc ggttcccaacgatcaaggcgagttacatgatcccccatgttgtgcaaaaaagcggttagctccttcggtcctccgatcgttgtcagaagt aagttggccgcagtgttatcactcatggttatggcagcactgcataattctcttactgtcatgccatccgtaagatgcttttctgtgactggtg agtactcaaccaagtcattctgagaatagtgtatgcggcgaccgagttgctcttgcccggcgtcaatacgggataataccgcgccacat agcagaactttaaaagtgctcatcattggaaaacgttcttcggggcgaaaactctcaaggatcttaccgctgttgagatccagttcgatgt aacccactcgtgcacccaactgatcttcagcatcttttactttcaccagcgtttctgggtgagcaaaaacaggaaggcaaaatgccgcaa aaaagggaataagggcgacacggaaatgttgaatactcatactcttcctttttcaatattattgaagcatttatcagggttattgtctcatgag cggatacatatttgaatgtatttagaaaaataaacaaataggggttccgcgcacatttccccgaaaagtgccacctgacgtcgctagctgt acaaaaaagcaggctttaaaggaaccaattcagtcgactggatccggtaccaaggtcgggcaggaa

pU6-pegRNA-Te:

gagggcctatttcccatgattccttcatatttgcatatacgatacaaggctgttagagagataattagaattaatttgactgtaaacacaaag atattagtacaaaatacgtgacgtagaaagtaataatttcttgggtagtttgcagttttaaaattatgttttaaaatggactatcatatgcttacc gtaacttgaaagtatttcgatttcttggctttatatatcttgtggaaaggacgaaacaccgagaccgaggtctcgcgcggttctatctagtt

acgcgttaaaccaactagaatttttttaagcttgggccgctcgaggtacctctctacatatgacatgtgagcaaaaggccagcaaaaggc
caggaaccgtaaaaaggccgcgttgctggcgtttttccataggctccgcccccctgacgagcatcacaaaaatcgacgctcaagtcag
aggtggcgaaacccgacaggactataaagataccaggcgtttccccctggaagctccctcgtgcgctctcctgttccgaccctgccgct
taccggatacctgtccgcctttctcccttcgggaagcgtggcgctttctcatagctcacgctgtaggtatctcagttcggtgtaggtcgttc
gctccaagctgggctgtgtgcacgaaccccccgttcagcccgaccgctgcgccttatccggtaactatcgtcttgagtccaacccggta
agacacgacttatcgccactggcagcagccactggtaacaggattagcagagcgaggtatgtaggcggtgctacagagttcttgaagt
ggtggcctaactacggctacactagaagaacagtatttggtatctgcgctctgctgaagccagttaccttcggaaaaagagttggtagct
cttgatccggcaaacaaaccaccgctggtagcggtggtttttttgtttgcaagcagcagattacgcgcagaaaaaaaggatctcaagaa
gatcctttgatcttttctacggggtctgacgctcagtggaacgaaaactcacgttaagggattttggtcatgagattatcaaaaaggatcttc
acctagatccttttaaattaaaaatgaagttttaaatcaatctaaagtatatatgagtaaacttggtctgacagttaccaatgcttaatcagtga
ggcacctatctcagcgatctgtctatttcgttcatccatagttgcctgactccccgtcgtgtagataactacgatacgggagggcttaccat
ctggccccagtgctgcaatgataccgcgagatccacgctcaccggctccagatttatcagcaataaaccagccagccggaagggccg
agcgcagaagtggtcctgcaactttatccgcctccatccagtctattaattgttgccgggaagctagagtaagtagttcgccagttaatagt
ttgcgcaacgttgttgccattgctacaggcatcgtggtgtcacgctcgtcgtttggtatggcttcattcagctccggttcccaacgatcaag
gcgagttacatgatcccccatgttgtgcaaaaaagcggttagctccttcggtcctccgatcgttgtcagaagtaagttggccgcagtgtta
tcactcatggttatggcagcactgcataattctcttactgtcatgccatccgtaagatgcttttctgtgactggtgagtactcaaccaagtcat
tctgagaatagtgtatgcggcgaccgagttgctcttgcccggcgtcaatacgggataataccgcgccacatagcagaactttaaaagtg
ctcatcattggaaaacgttcttcggggcgaaaactctcaaggatcttaccgctgttgagatccagttcgatgtaacccactcgtgcaccca
actgatcttcagcatcttttactttcaccagcgtttctgggtgagcaaaaacaggaaggcaaaatgccgcaaaaaagggaataagggcg
acacggaaatgttgaatactcatactcttcctttttcaatattattgaagcatttatcagggttattgtctcatgagcggatacatatttgaatgt
atttagaaaaataaacaaataggggttccgcgcacatttccccgaaaagtgccacctgacgtcgctagctgtacaaaaaagcaggcttt
aaaggaaccaattcagtcgactggatccggtaccaaggtcgggcaggaa

U6 promotor

gRNA scaffold

Tmpknot

Tevopreq1
